# Cardiac Structure Doses in Women Irradiated for Breast Cancer in the Past and Their Use in Epidemiological Studies

**DOI:** 10.1016/j.prro.2019.01.004

**Published:** 2019

**Authors:** Frances K. Duane, Paul McGale, Dorthe Brønnum, David J. Cutter, Sarah C. Darby, Marianne Ewertz, Sara Hackett, Per Hall, Ebbe L. Lorenzen, Kazem Rahimi, Zhe Wang, Samantha Warren, Carolyn W. Taylor

**Affiliations:** a Medical Research Council Population Health Research Unit, Nuffield Department of Population Health, University of Oxford, United Kingdom; b Clinical Trial Service Unit, Nuffield Department of Population Health, University of Oxford, United Kingdom; c Centre for Clinical Research, North Denmark Regional Hospital/Department of Clinical Medicine, Aalborg University, Hjørring, Denmark; d Department of Oncology, Odense University Hospital, Institute of Clinical Research, University of Southern Denmark, Denmark; e CRUK/MRC Oxford Institute for Radiation Oncology, Gray Laboratories, University of Oxford, United Kingdom; f Department of Medical Epidemiology and Biostatistics, Karolinska Institute, Stockholm, Sweden; g Department of Oncology, South General Hospital, Stockholm, Sweden; h Laboratory of Radiation Physics, Odense University Hospital, Odense, Denmark; i George Institute for Global Health, University of Oxford, Oxford, United Kingdom; j University of Birmingham NHS Foundation Trust, Birmingham, United Kingdom

## Abstract

**Purpose:**

Incidental cardiac exposure during radiation therapy may cause heart disease. Dose-response relationships for cardiac structures (segments) may show which ones are most sensitive to radiation. Radiation-related cardiac injury can take years to develop; thus, studies need to involve women treated using 2-dimensional planning, with segment doses estimated using a typical computed tomography (CT) scan. We assessed whether such segment doses are accurate enough to use in dose-response relationships using the radiation therapy charts of women with known segment injury. We estimated interregimen and interpatient segment dose variability and segment dose correlations.

**Methods and Materials:**

The radiation therapy charts of 470 women with cardiac segment injury after breast cancer radiation therapy were examined, and 41 regimens were identified. Regimens were reconstructed on a typical CT scan. Doses were estimated for 5 left ventricle (LV) and 10 coronary artery segments. Correlations between cardiac segments were estimated. Interpatient dose variation was assessed in 10 randomly selected CT scans for left regimens and in 5 for right regimens.

**Results:**

For the typical CT scan, interregimen segment dose variation was substantial (range, LV segments <1-39 Gy; coronary artery segments <1-48 Gy). In 10 CT scans, interpatient segment dose variation was higher for segments near field borders (range, 3-47 Gy) than other segments (range, <2 Gy). Doses to different left-anterior descending coronary artery (LADCA) segments were highly correlated with each other, as were doses to different LV segments. Also, LADCA segment doses were highly correlated with doses to LV segments usually supplied by the LADCA. For individual regimens there was consistency in hotspot location and segment ranking of higher-versus-lower dose.

**Conclusions:**

The scope for developing quantitative cardiac segment dose-response relationships in patients who had 2-dimensional planning is limited because different segment doses are often highly correlated, and segment-specific dose uncertainties are not independent of each other. However, segment-specific doses may be reliably used to rank segments according to higher-versus-lower doses.

## Introduction

Breast cancer radiation therapy reduces breast cancer mortality^1^, ^2^ but may increase the risk of ischaemic heart disease (IHD)^3^, ^4^ by causing macrovascular coronary artery disease or microvascular myocardial disease.^5^ Most evidence that links breast cancer radiation therapy with heart disease is based on women treated in previous decades with outdated techniques. A number of contemporary studies suggest that modern regimens pose a much-reduced risk of radiation-induced heart disease owing to improvements in radiation therapy techniques,^6^, ^7^, ^8^, ^9^ and in some cases a reduction in the prescribed doses.^10^, ^11^, ^12^ Nonetheless, radiation-induced heart disease is still likely to be relevant to subgroups of women such as those who cannot tolerate breathing adaptation, have an atypical anatomy, or are undergoing internal mammary chain irradiation.

Currently, doses to small regions, such as cardiac structures, in 3-dimensional computed tomography (CT)-based radiation therapy planning can be modified by changing beam angles or using a different technique. As a result, oncologists often have a choice as to which structures are exposed. The coronary arteries and myocardium have different structures and functions and may respond differently to radiation. Knowing if the dose-response relationship was steeper for radiation-related coronary artery disease or myocardial disease, or whether they were equally sensitive, would be useful to know. However, few studies to date have related coronary artery or left ventricle (LV) segment radiation doses to detailed cardiology information.^13^, ^14^

In a recent case-control study of 963 women who developed IHD after breast cancer radiation therapy, the best available predictor of IHD was mean heart dose,^3^ and coronary artery doses were not significantly associated with the rate of IHD events after the mean whole heart dose was taken into account. This may be because the coronary arteries and myocardium are equally sensitive to radiation. Alternatively, it may be due to the strong correlations between coronary artery and myocardial doses in breast cancer radiation therapy and the greater uncertainties in estimated coronary artery doses compared with whole heart doses.^15^, ^16^

Clinical cardiac disease often occurs years after exposure; thus, studies that relate cardiac doses to radiation-related injury inevitably need to be carried out in patients treated before the era of CT-based radiation therapy planning. Information on cardiac segment injury for these patients can be abstracted from angiogram and echocardiogram reports in their cardiology medical notes. Individual radiation therapy charts can be abstracted from oncology notes, but because these women did not receive CT-planning, segment doses need to be estimated retrospectively by reconstructing the regimens on a typical CT scan. How reliable these typical segment doses are, and whether they can be used in segment dose-response relationships, is unclear.

This study describes the estimation of cardiac segment doses for women who received 2-dimensional planned breast cancer regimens and subsequently developed segment damage. We describe the interregimen variation in segment doses for 41 regimens and interpatient variation in segment doses for 14 commonly used regimens. We considered how these segment doses may be used to assess associations between dose and segment injury. Our results were used to inform a separate study that related segment doses to sites of injury.^17^

## Methods and Materials

### Regimens

Regimens were identified from the radiation therapy charts of 470 women included in a population-based study of major coronary events with known location of segment injury after breast cancer radiation therapy.^3^, ^17^ The women were irradiated in Sweden between 1958 and 2001 or Denmark between 1978 and 2000. Radiation therapy charts included diagrams or photographs of the treatment fields, and sometimes dose-plans. Details on the surgery, target definition, field borders, target dose, applied total dose, dose per fraction, beam energy and use of shielding, wedges, and bolus were collected. Information was also collated from radiation therapy protocols.

### Contouring

Ten radiation therapy CT-planning scans were randomly selected from women irradiated at Odense University Hospital in Denmark in 2010. The treatment position was supine, with both arms above the head. The scan slice thickness was 3 mm, and intravenous contrast was not used. The whole heart, ventricles, and LV and coronary artery segments were contoured on all 10 scans using an atlas.^18^ To simulate a mastectomy, the breast was contoured and assigned a CT-value for air and 1 cm of tissue was retained above the pectoralis major muscle to account for residual subcutaneous tissue.

### Selection of typical computed tomography scan

**Fig. 1:**
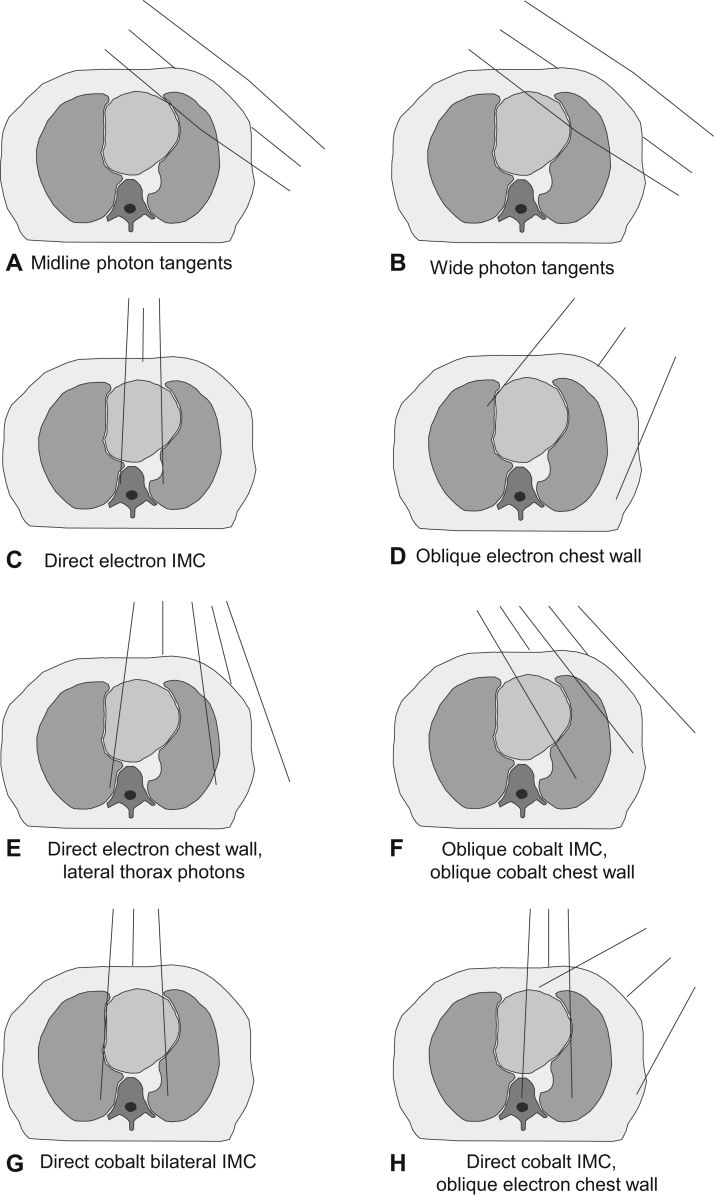
Radiation therapy fields used to treat women with breast cancer in Sweden (1958-2001) or Denmark (1978-2000). As is typical in radiation therapy planning, the patient's right is on the reader's left. Adapted from Taylor (2007).^23^

The 2 most common left-sided regimens for the women in the study were identified and reconstructed on all 10 CT scans, which were a midline tangential regimen used after breast conserving surgery (Fig. 1A; Table E1 [field arrangement 3]; available online at https://doi.org/10.1016/j.prro.2019.01.004) and a direct electron chest wall regimen used after mastectomy (Fig. 1E; Table E1 [field arrangement 7]). Whole heart and cardiac substructure doses were collated, as were whole heart volume, chest wall separation, sternal length, and Haller index (ie, ratio of height between anterior spine and posterior sternum to transverse width of the chest). The scan with the mean heart doses closest to average for both techniques, and which was not atypical for any of the anatomical factors examined, was selected as the typical CT scan (Table E2; available online at https://doi.org/10.1016/j.prro.2019.01.004).

### Reconstruction and dose calculation

All identified regimens were reconstructed on this typical CT scan using 3-dimensional treatment planning (Varian Eclipse Treatment Planning System, version 10.0.39). Lines were drawn on the body surface to represent clinical landmarks used to plan 2-dimensional radiation therapy in previous decades. Field borders, gantry angles, and custom blocks^19^ were guided by these clinical surface markings and digitally reconstructed radiographs.

The dose calculation algorithms were an analytical anisotropic algorithm for photon plans, Monte Carlo for electron plans, and pencil beam for cobalt plans. If photon beam energies were unavailable, they were created using mixed energy beams. The dose was calculated using the 0.1 cm calculation volume grid for all, except the cobalt regimens where the minimum grid was 0.25 cm. Dose-volume histograms (DVHs) were exported for each cardiac segment with dose-bins of 0.1%.

Orthovoltage regimens were reconstructed by manual planning. Field borders were defined using CT-based 3-dimensional virtual simulation. Ten axial CT images (CT slice spacing: 1.2 cm) spanning the heart were printed and scaled up to life size. Isodose charts were superimposed onto each CT slice and used to map dose onto cardiac segments. The isodose shift method was used to correct for lung in the field(s) and standoff at the body surface (isodose shift factor: 0.8). The proportions of each cardiac segment included within the isodose lines were calculated. DVHs were plotted and mean segment doses calculated.

For all regimens, DVHs were used to calculate the mean doses in equivalent 2 Gy fractions, which was separately calculated for each dose bin in each DVH using:

nd[(d+α/β)/(2+α/β)]

where n = number of fractions, d = mean dose to the cardiac structure per fraction (Gy), and α/β = 2 Gy.^20^, ^21^

### Interpatient dose variation

The effect of interpatient variability in anatomy on cardiac segment doses was investigated in 14 regimens (with different field arrangements) from the 2 most common technique categories. Eight of these 14 regimens (4 left-sided and 4 right-sided) were tangential (Table E1; Table 1 [techniques 1-4]) and 6 (3 left- and 3 right-sided) were anterior electron regimens (Table E1; Table 1 [techniques 5-7]). The left regimens were reconstructed on all 10 CT scans, and the right regimens on only 5 scans because interpatient variability in cardiac dose is usually lower from right radiation therapy.^22^

**Table 1:** Mean radiation therapy doses to myocardial structures from left-sided breast cancer radiation therapy regimens used in Sweden (1958-2001) or Denmark (1978-2000)

Radiation therapy regimen∗						Mean cardiac doses (Gy)†							
Country	Median Year	Medial Border	Field arrangement‡§	Usual beam energy	Dose (100%) Gy¶	Whole	Ventricles		Left ventricular myocardial segments				
						Heart	Left	Right	Apex	Lateral	Inferior	Septal	Anterior
Tangential													
Sweden	1959	Midline	Tangents, divergent (a)	170 kV	10.5	2	3	3	8	3	1	3	3
	1975	6 cm contra	Wide tangents, divergent (b)	Co^60^	45.0	**11.3**	**13.6**	**18.3**	**35.7**	**6.4**	**2.0**	**16.3**	**18.8**
	1982	Midline	Tangents, divergent (a)	Co^60^	50.0	5.3	7.6	4.7	33.2	3.0	1.3	5.3	9.2
	1990	Midline	Tangents, divergent (a) (1)	6 MV	50.0	4.8	7.2	3.6	37.1	2.3	1.0	4.2	7.7
Denmark	1981	3 cm contra	Wide tangents, divergent (b) (2)	6 MV	40.7	**9.1**	**12.5**	**13.0**	**38.8**	**5.7**	**1.3**	**12.8**	**18.2**
	1982	2 cm contra	Wide tangents (McWhirter) (b)	250 kV	36.0	**11**	**13**	**13**	**31**	**12**	**6**	**14**	**18**
	1994	Midline	Tangents, block posteriorly (a) (3)	8 MV	48.0	4.0	6.0	3.0	31.2	2.0	0.6	3.3	7.6
	1998	3 cm contra	Partially wide tangents, block posteriorly (i) (4)	8 MV	48.0	**4.9**	**5.9**	**6.1**	**22.5**	**2.6**	**0.7**	**4.7**	**11.7**
Anterior electron or orthovoltage													
Sweden	1960	1 cm contra	Direct IMC (1-field) (c)	170 kV	28.0	9	7	11	5	5	7	13	7
	1963	Midline	Direct IMC (2-fields) (c) (j) (5)	12 MeV	40.0	3.7	0.8	8.3	0.5	0.4	0.4	2.1	1.5
	1963	1 cm contra	Direct chest wall (4 fields) (k)	170 kV	20.0	7	5	8	3	4	5	9	6
	1974	1 cm contra	Oblique chest wall (d) (6)	12 MeV	47.8	**9.4**	**8.6**	**14.4**	**28.7**	**5.2**	**0.7**	**9.1**	**19.1**
Denmark	1981	Midline	Direct chest wall (1-field)/lat thorax, SCF, axilla (n)	100 kV/8 MV	36.0/50.0	10	9	11	6	7	4	11	21
	1982	1 cm contra	Direct chest wall (1-field)/lat thorax, SCF, axilla (e) (m) (7)	9 MeV/8 MV	51.8/51.7	6.7	4.6	9.3	9.6	3.1	2.0	4.2	14.4
	1987	1 cm contra	Direct chest wall (2-fields)/lat thorax, SCF, axilla (l)	9 MeV/6 MeV/8 MV	54.0/50.0/54.0	5.4	3.2	8.5	4.0	3.3	2.4	3.1	5.0
	1991	Midline	Oblique chest wall (d)	12 MeV	51.8	**10.2**	**9.4**	**15.6**	**31.2**	**5.6**	**0.8**	**9.8**	**20.7**
Anterior megavoltage													
Sweden	1969	4.5 cm contra	Oblique IMC/oblique chest wall (f)	Co^60^/Co^60^	36.0/32.0	21.9	23.1	27.6	27.1	20.3	19.6	25.6	23.8
	1974	4.5 cm contra	Direct bilateral IMC (g)‖	Co^60^	40.0	21.7	12.8	30.0	1.8	4.4	21.1	24.1	5.8
	1983	Midline	Direct IMC/oblique chest wall (h)	Co^60^/9 MeV	50.6/48.3	20.8	20.1	28.6	10.5	9.9	23.1	30.6	18.4
Cobalt chain∗∗													
Sweden	1963	Midline	Cobalt chain long (overlapping fields) (o)	Co^60^	7.0	4	6	4	6	2	2	4	6
	1966	Midline	Cobalt chain short (overlapping fields) (p)	Co^60^	7.0	<1	<1	<1	<1	<1	<1	<1	<1

*Abbreviations:* Co^60^ = cobalt 60; contra = contralateral; IMC = internal mammary chain; kV = kilovoltage; lat = lateral; MeV = mega electron-volts; MV = megavoltage; SCF = supraclavicular fossa

Bold tangential regimens are *wide* tangents, others are *midline* tangents. Bold anterior electron or orthovoltage regimens are *oblique* electron fields, others include *direct* fields.

∗ For further details on the radiation therapy regimens, see Table E1.

† Mean cardiac doses estimated using manual planning (ie, orthovoltage and cobalt chain) are given to the nearest Gy.

‡ Regimens a through p are illustrated in Figures 1 and E1 (available online at https://doi.org/10.1016/j.prro.2019.01.004).

§ Regimens 1 through 7 were reconstructed on 10 scans to study the effect of patient anatomy on segment doses (Fig. 3, Fig. 4, Fig. 5).

¶ Usual total dose (100%) to the target tissues. For direct regimens, this was the D_max_, and for tangential regimens the dose delivered to the center of the breast/chest wall, except for orthovoltage tangents where the total dose was the skin dose at the surface of the breast.

‖ Cardiac doses are the same for left- and right-sided breast cancers because the same field was used for both.

∗∗ For a description of cobalt chain, see Taylor (2009).^23^

For each regimen, interpatient variability in mean doses was calculated as the difference between the highest and lowest mean doses recorded for each cardiac segment. Interpatient variability in hotspot doses was calculated as the difference between the highest and lowest hotspot doses recorded for each cardiac segment. The hotspot doses recorded included D2cc (minimum dose covering the hottest 2.0 cc) of the ventricular myocardium (right ventricle [RV] and LV combined) and D0.5cc (minimum dose covering the hottest 0.5 cc) of the main coronary arteries (left main coronary artery, left-anterior descending coronary artery [LADCA], right coronary artery [RCA], and circumflex coronary artery [Cx] combined).

### Correlations

Correlation analyses between cardiac segment and whole heart doses, and between coronary artery segment and LV segment doses, were performed using STATA, version 13.2 (StataCorp, College Station, TX).

## Results

A total of 41 regimens were identified from 470 radiation therapy charts, including 20 regimens for left breast cancer, 20 for right cancer, and 1 that was the same for left and right cancers (Fig. 1, Fig. E1, Table E1).

### Tangential regimens

**Fig. 2:**
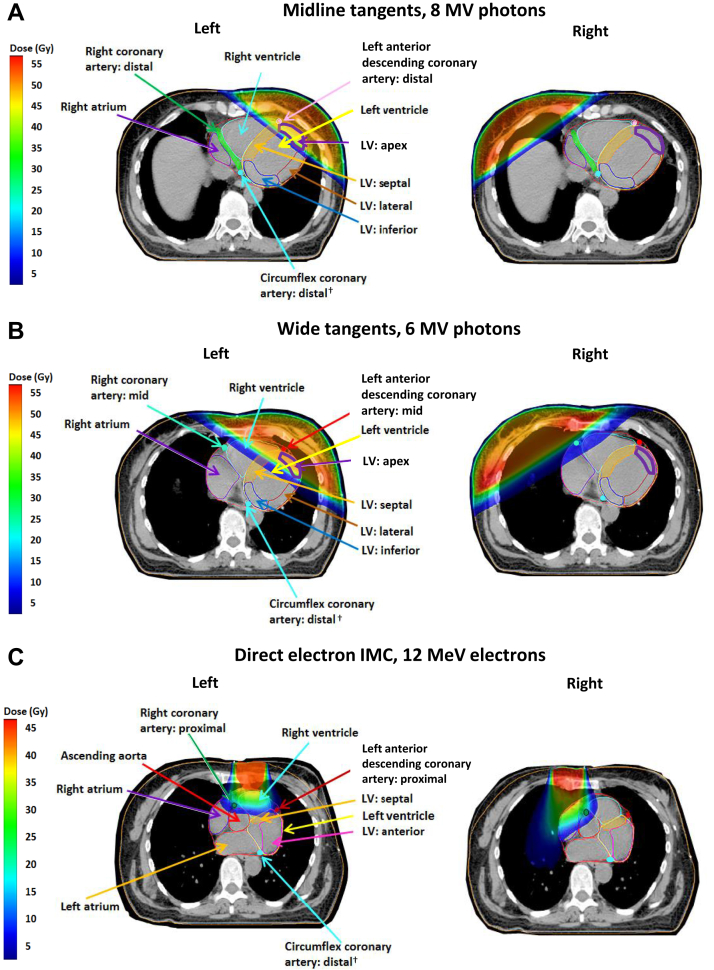
Spatial distribution of cardiac dose from breast cancer tangential and direct electron radiation therapy regimens used to treat women with breast cancer in Sweden (1958-2001) or Denmark (1978-2000). Left regimens A-C are illustrated in Fig. 1. ^†^ The main circumflex coronary artery has only 2 segments: Proximal and distal. *Abbreviations:* MV = megavoltage; MeV = mega electron-volts.

For left megavoltage tangential radiation therapy, cardiac doses were determined mainly by the position of the medial border and divergence of the posterior border (Tables 1, 2, E3, and E4 [available online https://doi.org/10.1016/j.prro.2019.01.004], Fig. 2). For left midline megavoltage tangents, the mean doses were 6.0 Gy to 7.6 Gy for LV and 3.0 Gy to 4.7 Gy for RV (Table 1, Fig. 2A). For left wide megavoltage tangents with a divergent posterior field border, both ventricles received ≥12.5 Gy, but for the partially wide tangents, both ventricles received <6.1 Gy (Table 1, Fig. 2B). For the left megavoltage tangents, the mean individual LV segment doses varied substantially (range, 0.6-38.8 Gy). The LV apex received the highest doses of ≥22.5 Gy. The anterior LV segment received ≥11.7 Gy from the wide divergent and partially wide megavoltage tangents but ≤9.2 Gy from the midline megavoltage tangents. The LV septal segment received ≥12.8 Gy from the wide divergent megavoltage tangents but ≤5.3 Gy from partially wide and midline megavoltage tangents. The lateral and inferior segments were further from the fields and received ≤6.4 Gy from all left megavoltage tangents.

**Table 2:** Mean radiation therapy doses to coronary arterial structures from left-sided breast cancer radiation therapy regimens used in Sweden (1958-2001) or Denmark (1978-2000)

Radiation therapy regimen∗						Mean coronary artery doses (Gy)†												
Country	Median Year	Medial Border	Field arrangement‡§	Usual beam energy	Dose (100%) Gy¶	Left	Left anterior descending				Right					Circumflex		
						Main	Whole	Prox	Mid	Dist	Whole	Prox	Mid	Dist	Pd	Whole	Prox	Dist
Tangential																		
Sweden	1959	Midline	Tangents, divergent (a)	170 kV	10.5	1	8	6	9	9	2	2	1	1	2	1	2	1
	1975	6 cm contra	Wide tangents, divergent (b)	Co^60^	45.0	**3.8**	**35.7**	**28.3**	**39.6**	**38.8**	**5.7**	**7.7**	**8.2**	**2.0**	**4.9**	**1.8**	**3.0**	**1.6**
	1982	Midline	Tangents, divergent (a)	Co^60^	50.0	1.9	31.7	11.0	41.8	41.2	1.6	1.8	1.6	1.2	1.7	1.3	1.8	1.2
	1990	Midline	Tangents, divergent (a) (1)	6 MV	50.0	1.5	34.1	8.6	46.2	46.1	1.2	1.4	1.1	0.8	1.3	0.9	1.5	0.8
Denmark	1981	3 cm contra	Wide tangents, divergent (b) (2)	6 MV	40.7	**1.8**	**36.2**	**25.2**	**41.8**	**40.4**	**2.4**	**2.3**	**2.2**	**1.3**	**3.5**	**1.1**	**1.7**	**1.0**
	1982	2 cm contra	Wide tangents (McWhirter) (b)	250 kV	36.0	**4**	**33**	**24**	**37**	**36**	**7**	**9**	**7**	**5**	**7**	**5**	**8**	**5**
	1994	Midline	Tangents, block posteriorly (a) (3)	8 MV	48.0	1.1	33.1	8.9	46.0	43.4	0.8	1.0	0.7	0.4	0.9	0.6	1.1	0.5
	1998	3 cm contra	Partially wide tangents, block posteriorly (i) (4)	8 MV	48.0	**2.0**	**33.7**	**18.9**	**48.0**	**32.8**	**1.8**	**2.7**	**2.6**	**0.6**	**1.2**	**0.7**	**1.7**	**0.6**
Anterior electron or orthovoltage																		
Sweden	1960	1 cm contra	Direct IMC (1-field) (c)	170 kV	28.0	16	10	14	11	7	10	18	11	11	3	10	14	9
	1963	Midline	Direct IMC (2-fields) (c) (j) (5)	12 MeV	40.0	4.6	3.4	7.0	2.9	0.5	4.7	13.1	4.0	0.4	0.2	0.8	1.8	0.6
	1963	1 cm contra	Direct chest wall (4 fields) (k)	170 kV	20.0	12	7	10	8	5	7	13	8	8	2	7	10	6
	1974	1 cm contra	Oblique chest wall (d) (6)	12 MeV	47.8	**8.3**	**34.4**	**30.2**	**36.3**	**36.3**	**6.0**	**9.4**	**11.5**	**1.0**	**1.4**	**1.4**	**4.9**	**0.9**
Denmark	1981	Midline	Direct chest wall (1-field)/lat thorax, SCF, axilla (n)	100 kV/8 MV	36.0/50.0	21	14	28	19	1	8	18	11	3	1	11	21	9
	1982	1 cm contra	Direct chest wall (1-field)/lat thorax,SCF, axilla (e) (m) (7)	9 MeV/8 MV	51.8/51.7	4.1	30.2	35.4	46.2	8.7	6.5	11.3	10.9	2.6	0.8	2.9	3.8	2.7
	1987	1 cm contra	Direct chest wall (2-fields)/lat thorax, SCF, axilla (l)	9 MeV/6 MeV/8 MV	54.0/50.0/54.0	3.5	13.5	14.6	17.7	7.5	5.6	10.8	7.4	2.0	1.4	2.8	3.4	2.7
	1991	Midline	Oblique chest wall (d)	12 MeV	51.8	**9.0**	**37.3**	**32.8**	**39.4**	**39.3**	**6.5**	**10.2**	**12.5**	**1.1**	**1.5**	**1.6**	**5.3**	**1.0**
Anterior megavoltage																		
Sweden	1969	4.5 cm contra	Oblique IMC/oblique chest wall (f)	Co^60^/Co^60^	36.0/32.0	25.1	28.1	27.1	28.4	28.6	25.0	29.0	29.0	19.5	21.3	19.3	22.5	18.8
	1974	4.5 cm contra	Direct bilateral IMC (g)‖	Co^60^	40.0	28.1	4.0	8.2	1.8	2.3	28.0	32.4	33.4	26.0	21.0	21.7	23.9	21.4
	1983	Midline	Direct IMC/oblique chest wall (h)	Co^60^/9 MeV	50.6/48.3	36.1	31.8	36.4	29.3	28.0	12.2	22.7	2.5	17.9	8.5	27.8	33.4	26.8
Cobalt chain∗∗																		
Sweden	1963	Midline	Cobalt chain long (overlapping fields) (o)	Co^60^	7.0	7	6	8	7	4	4	7	2	2	2	2	2	2
	1966	Midline	Cobalt chain short (overlapping fields) (p)	Co^60^	7.0	1	<1	<1	<1	<1	<1	<1	<1	<1	<1	<1	<1	<1

*Abbreviations:* contra = contralateral; Co^60^ = cobalt 60; dist = distal; IMC = internal mammary chain; kV = kilovoltage; lat = lateral; MeV = mega electron-volts; MV = megavoltage; prox = proximal; pd = posterior descending; SCF = supraclavicular fossa

Bold tangential regimens are *wide* tangents, others are *midline* tangents. Bold anterior electron or orthovoltage regimens are *oblique* electron fields, others include *direct* fields.

∗ For further details on the radiation therapy regimens see Table E1.

† Mean cardiac doses estimated using manual planning (ie, orthovoltage and cobalt chain) are given to nearest Gy.

‡ Regimens a through p are illustrated in Figure 1 and E1 (available online at https://doi.org/10.1016/j.prro.2019.01.004).

**Fig. 3:**
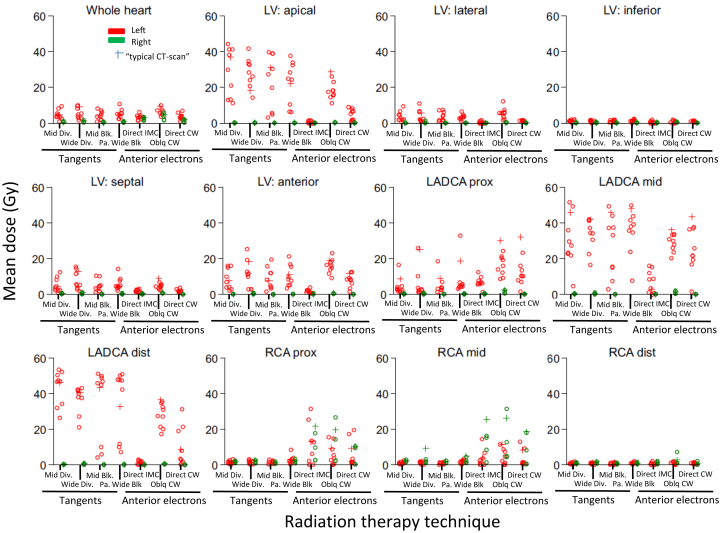
Patient-to-patient variability in mean radiation dose to the whole heart, and left ventricular and coronary arterial segments in women receiving tangential or anterior electron regimens for breast cancer in Sweden (1958-2001) or Denmark (1978-2000). For further details on regimens 1 through 7, see Table E1. Regimen definitions: Tangents (1) Mid. Div, midline divergent, (50 Gy/25 fractions); (2) Wide Div, wide divergent, medial border 3 cm contralateral, (41 Gy/22 fractions); (3) Mid. Blk, midline, blocked posterior border tapered inferiorly around breast (48 Gy/24 fractions); (4) Pa. Wide Blk., partially wide, medial border 3 cm contralateral, blocked posterior border tapered inferiorly below 5th rib (48 Gy/28 fractions). Anterior electron (5) Direct IMC: direct internal mammary chain (40 Gy/10 fractions); (6) Oblq CW: oblique chest wall (48 Gy/26 fractions); and (7) Direct CW: direct chest wall (52 Gy/24 fractions). *Abbreviations:* LADCA = left anterior descending coronary artery; LV = left ventricle; RCA = right coronary artery.

**Fig. 4:**
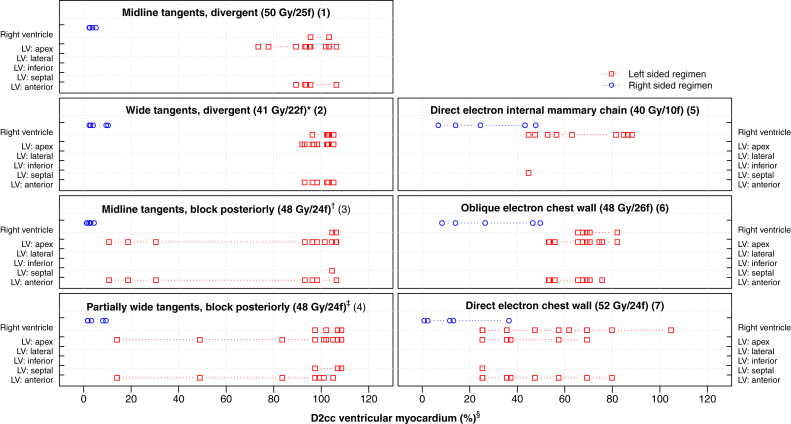
D2cc (%) hotspot doses to the ventricular myocardium (right and left ventricles combined) in women receiving tangential or anterior electron regimens for breast cancer in Sweden (1958-2001) or Denmark (1978-2000). For further details on regimens 1-7, see Table E1 (available online at https://doi.org/10.1016/j.prro.2019.01.004). ^∗^ Medial border: 3 cm contralateral. ^†^ Block tapered inferiorly around the breast. ^‡^ Block tapered inferiorly below the fifth rib, medial border: 3 cm contralateral. ^§^ Doses are shown in percent rather than Gy to enable a comparison based on patient anatomy rather than differing target dose. The spatial location of the D2cc ventricular myocardial hotspot was identified for each regimen/computed tomography combination using the dose range selection tool on the treatment planning system to highlight the voxels within the volume receiving this dose. Some hotspot volumes spanned >1 structure and were not always contiguous. *Abbreviations:* LV = left ventricle; f = fractions.

**Fig. 5:**
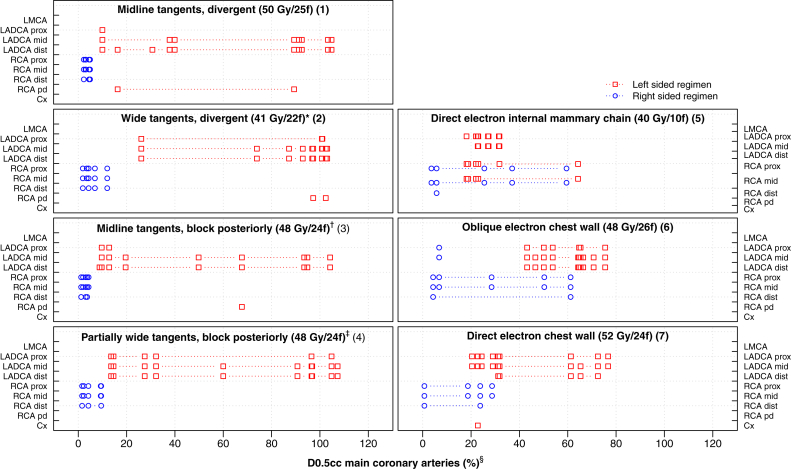
D0.5cc (%) hotspot doses to the main coronary arteries (left main coronary artery, LADCA, RCA and Cx combined) in women receiving tangential or anterior electron regimens for breast cancer in Sweden (1958-2001) or Denmark (1978-2000). For further details on regimens 1-7, see Table E1 (available online at https://doi.org/10.1016/j.prro.2019.01.004). ^∗^Medial border: 3 cm contralateral ^†^ Block tapered inferiorly around breast. ^‡^ Block tapered inferiorly below the fifth rib, medial border: 3 cm contralateral ^§^ Doses are shown in percent rather than Gy to enable a comparison based on patient anatomy rather than differing target dose. The spatial location of the D0.5cc main coronary arteries hotspot was identified for each regimen and computed tomography combination using the dose range selection tool on the treatment planning system to highlight the voxels within the volume receiving this dose. Some hotspot volumes spanned >1 structure and were not always contiguous. *Abbreviations:* LMCA = left main coronary artery; LADCA = left anterior descending coronary artery; RCA = right coronary artery; Cx = circumflex coronary artery; f = fractions; IMC = internal mammary chain; prox = proximal; dist = distal; pd = posterior descending.

§ Regimens 1 through 7 were reconstructed on 10 scans to study the effect of patient anatomy on segment doses (Fig. 3, Fig. 4, Fig. 5).

¶ Usual total dose (100%) to the target tissues. For direct regimens this was the D_max_, and for tangential regimens this was the dose delivered to the center of the breast/chest wall, except for orthovoltage tangents where the total dose was the skin dose at the surface of the breast.

‖ Cardiac doses are the same for left- and right-sided breast cancer as the same field was used for both.

∗∗ For a description of cobalt chain see, Taylor (2009).^23^

The mean doses to the coronary artery segments in the left megavoltage tangential radiation therapy varied from 0.4 Gy to 48.0 Gy. For all megavoltage tangents, the LADCA mid- and distal segments received the highest doses of ≥32.8 Gy (Table 2, Fig. 2). The LADCA proximal received ≥18.9 Gy from the wide divergent and partially wide megavoltage tangents and 8.6 Gy to 11.0 Gy from the midline megavoltage tangents. The RCA and Cx segments were further from the fields and received 0.4 Gy to 8.2 Gy.

For orthovoltage midline tangents, the prescribed dose was only 10.5 Gy, and all cardiac segments received ≤9 Gy (Tables 1, 2, E3 and E4). For McWhirter orthovoltage wide-tangential radiation therapy (36.0 Gy prescribed dose), whole LV and LADCA doses were similar to those for megavoltage wide tangents, but the RCA and Cx doses were higher owing to scattered radiation from orthovoltage beams.

For all right tangential regimens, the RV received higher mean doses (0.5-7.0 Gy) than the LV (0.1-3.0 Gy; Table E3, Fig. 2). The coronary artery segments all received ≤4 Gy, except for the RCA segments, which received 0.3 to 32.0 Gy from right megavoltage wide tangents (Table E4, Fig. 2).

### Anterior electron or orthovoltage regimens

For anterior electron or orthovoltage radiation therapy, the doses depended on the field borders, beam energy, and whether the beam was direct or oblique. For all regimens, the RV received higher doses than the LV because of its proximity to the anterior fields (left regimens: RV 8.0-15.6 Gy and LV 0.8-9.4 Gy; and right regimens: RV 1.9-6.0 Gy and LV 0.2-1.0 Gy; Table 1, Fig. 2, Table E3). The left oblique beams were angled toward the LV and gave higher LV doses (range, 8.6-9.4 Gy) than the left direct beams (range, 0.8-9.0 Gy). For the left oblique beams, the LV apex received the highest doses (range, 28.7-31.2 Gy). For the left direct beams, the anterior or septal LV segments received the highest doses (LV anterior: 1.5-21.0 Gy; LV septal: 2.1-13.0 Gy). Right-anterior electron or orthovoltage regimens delivered ≤5.0 Gy to all LV segments (Table E3).

The coronary artery segments closest to the fields received the highest doses. For the left regimens, these were the LADCA and RCA proximal and mid segments (range, 2.9-46.2 Gy; Table 2), and for the right regimens, the RCA proximal and mid segments (range, 12.7-28.5 Gy; Table E4).

### Anterior megavoltage regimens

Five anterior megavoltage regimens were used: 2 for left cancer, 2 for right cancer, and 1 regimen was the same in the left and right cancers. Most segments were in the radiation therapy fields and received >20 Gy (Fig. 1F-H, Tables 1, 2, E3, and E4).

### Cobalt chain regimens

Cobalt chain radiation therapy involved small, rectangular, overlapping cobalt fields in a vertical line along the internal mammary chain.^23^ The short cobalt chain was above the level of the heart, and all segments received <1 Gy (Tables 1, 2, E3, and E4). The long cobalt chain covered part of the heart and delivered <1 Gy to 10 Gy to the cardiac segments.

### Interpatient variation

Interpatient variability in the mean doses ranged from <1 Gy to 2 Gy for segments distant from the fields (Fig. 3). For segments near the fields, variability ranged from 3 Gy to 47 Gy for the left and 3 Gy to 27 Gy for the right regimens.

For LV segments in the left tangents, the LV apex, lateral, septal, and anterior segments were near field edges, which resulted in dose variability of 8 Gy to 37 Gy (Fig. 2, Fig. 3). For the LV inferior segment, the dose varied by <6 Gy. For the left anterior electrons, the LV segment dose variability was <17 Gy. For the right tangents and right-anterior electrons, the LV segment dose variability was <1 Gy.

For coronary artery segments and left tangents, dose variability for LADCA segments ranged from 14 Gy to 47 Gy (Fig. 2, Fig. 3). For coronary arterial segments further from the fields, the dose variation was 1 Gy to 11 Gy. For the right tangents, the dose variability was <8 Gy. For the left-anterior electrons, the LADCA segments and RCA proximal and mid segments were close to the fields, and for right-anterior electrons, the RCA proximal and mid segments were close to the fields. Dose variability for these segments ranged from 3 Gy to 41 Gy. For arterial segments further from the fields, the dose variability was <6 Gy.

Hotspot doses were located in the LV apex, LV anterior, and LADCA segments for most left regimens, and in the RV, RCA proximal, and RCA mid segments for most right regimens (Figs. 4 and 5). The order of the segments according to higher-versus-lower doses was the same as in the typical CT scan for 91% of LV segment and regimen combinations, 91% of whole coronary artery and regimen combinations, and 81% of coronary artery segment and regimen combinations (Tables E5 and E6; available online at https://doi.org/10.1016/j.prro.2019.01.004).

### Correlations between cardiac segments

**Fig. 6:**
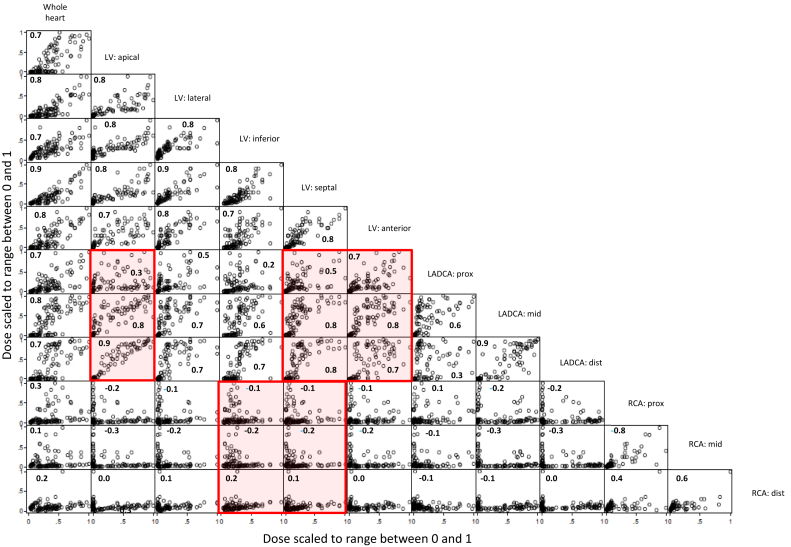
Correlations between mean radiation doses to the whole heart and different cardiac segments for women receiving tangential or anterior electron regimens for breast cancer in Sweden (1958-2001) or Denmark (1978-2000). To display correlations, all doses are scaled to range between 0 and 1. Each panel includes 105 points to show all estimated mean doses from 7 left-sided regimens reconstructed on 10 computed tomography scans and 7 right-sided regimens reconstructed on 5 computed tomography scans. Panels that show correlations between left-anterior descending coronary artery and right coronary artery segment doses and doses to the left ventricular (LV) segments usually supplied by the left anterior descending coronary artery (LV: apical, LV: septal, LV: anterior) and right coronary artery (LV: inferior, LV: septal) are highlighted.

The doses to all LV and LADCA segments were highly correlated with doses to the whole heart (range of correlation coefficients, 0.7-0.9; all *P* < .0001; Fig. 6). Correlations between the RCA segments and whole heart were much lower (range of correlation coefficients, 0.1-0.3; *P* = .008- .25). LADCA mid and distal segment doses were highly correlated with doses to the LV segments usually supplied by the LADCA: LV apex, and anterior and septal segments (range of correlation coefficients, 0.7-0.9; all *P* < .0001), but the RCA segment doses showed little correlation with doses to the LV segments usually supplied by the RCA: LV inferior, and septal segments (range of correlation coefficients, -0.1 to 0.2; *P* = .04 to .5).

## Discussion

Cardiac segment radiation doses from 41 breast cancer regimens, estimated retrospectively using information from radiation therapy charts, varied substantially. For most regimens, certain segments received >20 Gy, but others received <1 Gy. Different segments received high doses from different regimens. Such variability provides potential opportunities for the assessment of the effects of different doses to individual segments.

Many years hence, cardiac doses based on patient-specific CT-planning scans may be available to study long-term adverse effects. At the present time, however, most patients who developed clinical heart disease after radiation therapy were irradiated before the 2000s and did not receive CT-based dosimetry planning. Therefore, estimating cardiac doses using a typical CT scan is necessary. This method previously enabled the derivation of a linear dose-response relationship for radiation-related IHD (expressed as percentage increase in IHD rate per Gy mean whole heart dose). This dose response relationship has been validated in 2 independent studies.^24^, ^25^

When using the typical CT-scan method, cardiac doses actually received by individual patients vary on the estimated dose, principally owing to interpatient differences in anatomy. In our study, the effect of interpatient differences in anatomy varied by regimen and segment and was the greatest for segments that were near the field edges, and thus close to the high-dose gradient at the anterior aspect of the heart. This type of error is known as a Berkson error.^26^ For studies in which the outcome measure of interest is a continuous variable, Berkson errors do not bias the slope of the estimated dose response. In the present case, where the outcome measure is binary, Berkson errors in estimated doses may result in small biases to estimated dose-response relationships.^26^ This is in contrast with classic measurement errors, which can lead to considerable attenuation of derived dose-response relationships.

Doses to different segments were highly correlated, which would make investigating whether myocardial injury resulted from exposure of the myocardium causing disruption of the microvasculature or from exposure of an artery (eg, LADCA causing arterial occlusion and downstream myocardial ischaemia) difficult. Interestingly, RCA segment doses were not correlated with doses to the LV segments that are usually supplied by the RCA, which may provide an opportunity to differentiate between myocardial injury caused by microvascular injury versus that caused by macrovascular injury. Differences between doses actually delivered to individual segments and surrogate doses assigned based on the typical CT scan (owing to positional and anatomical uncertainties) will also be correlated. They may be positively correlated, for example, two adjacent segments may move into or out of the high dose region together such as the LV apex and distal LAD coronary artery in left-tangential radiotherapy (Fig. 2A). Or, they may be negatively correlated, for example, if patient position changed slightly during left direct electron IMC radiotherapy, the proximal LAD might move out of the field, and the proximal RCA move into the field (Fig. 2C).

In cardiac radiation dosimetry, high correlations between the estimated segment doses limit the ability to derive meaningful quantitative associations between doses and injury to specific cardiac segments. This difficulty is compounded by the fact that differences between the actual and estimated doses are also strongly correlated. Therefore, using these to derive quantitative dose-response relationships would be inappropriate. However, typical CT-scan doses consistently indicated whether a particular regimen typically gave a high, medium, or low dose to a segment (Tables E5 and E6), and our segment dose rankings were consistent with those in other publications.^13^, ^27^, ^28^, ^29^ There was also consistency in the locations of hotspots within the ventricular myocardium and main coronary arteries for each regimen (Figs. 4 and 5). Therefore, segment-specific doses may be used to rank segments by higher-versus-lower doses. Subsequently, these rankings may be related to the risks of cardiac segment injury in patients who received radiation therapy in the past to ascertain if segments that receive higher doses have a higher risk of injury. These findings may also be relevant to studies of women receiving contemporary radiation therapy.

Our study has several strengths. First, detailed information was collated on regimens from several sources, including individual radiation therapy charts for 470 women. Second, doses to coronary artery segments were estimated rather than whole coronary arteries. Arteries are long, thin structures that track in different directions around the heart. Segment doses may be more meaningful because generally only 1 or 2 segments of an artery receive a substantial dose, and other segments receive only a scattered dose. Third, we verified that the segments described were those referred to by the cardiologists when reporting the location of the cardiac injury, so that segment doses could be directly related to the location of the damage.

Our mean whole heart doses are consistent with those of other published estimates for similar regimens (Table E7; available online at https://doi.org/10.1016/j.prro.2019.01.004). Dosimetric uncertainties are larger for cardiac segment doses than for mean whole heart doses because segment doses are more sensitive to interpatient anatomical variability than whole heart doses. Our estimated cardiac doses are subject to several other unavoidable sources of uncertainty that are common to all radiation therapy CT-planning studies, including errors in contouring,^18^, ^30^ dose calculation algorithms,^31^ setup,^32^, ^33^ and errors caused by cardiac and respiratory motion during treatment.^34^, ^35^, ^36^

## Conclusions

Cardiac segment-specific doses may be used to rank segments by higher-versus-lower doses in epidemiological studies relating cardiac structure injury to radiation dose.^17^ However, the scope for developing quantitative dose-response relationships for cardiac segment injury based on information from the radiation therapy charts of patients treated before the era of 3-dimensional CT planning is limited because different segment doses are often highly correlated, and segment-specific dose uncertainties are not independent of each other.

## Supplementary Data

Tables E1-E11 and Fig. E1
