# Supplementary material for: Cardiac Structure Doses in Women Irradiated for Breast Cancer in the Past and Their Use in Epidemiological Studies
Source: Pract Radiat Oncol. 2019 May-Jun;9(3):158–71. doi: 10.1016/j.prro.2019.01.004 (PMC6493043; doi:10.1016/j.prro.2019.01.004)
Supplement: Tables E1-E11 and Fig. E1 [file mmc1.pdf]

Table E1. Radiotherapy regimens received by 470 women with breast cancer in Sweden (1958-2001) or Denmark (1978-2000).*

Country	*Median year*	*Field arrangement†‡*	*Number of women*	*Usual beam energy*	*Target(s)§*	*Dose (100%) Gy¶*	*Dose/ fraction*	*Field borders*	*Comments*
Tangential (n=185)									
Sweden	1959	Tangents (a)	9	170 kV	Breast	10.5||	3.5	Superior: 2nd costal cartilage<br>Inferior: 1 cm below inframammary fold<br>Medial: midline<br>Lateral: mid-axillary line	Posterior border divergent<br>Opposing symmetrical fields
	1975	Wide tangents (b)	42	Co^{60}	Breast or chest wall, IMC	36.0-65.0	1.4-2.5	Superior: 2nd costal cartilage<br>Inferior: 1 cm below inframammary fold<br>Medial: 6 cm from midline contra<br>Lateral: mid-axillary line	Posterior border divergent<br>Opposing symmetrical fields<br>Collimator not angled<br>Fixed SSD 80 cm
	1982	Tangents (a)	15	Co^{60}	Breast or chest wall	45.0-52.5	1.8-2.0	Superior: 2nd costal cartilage<br>Inferior: 1 cm below inframammary fold<br>Medial: midline<br>Lateral: mid-axillary line	Posterior border divergent<br>Opposing symmetrical fields<br>Collimator not angled<br>Fixed SSD 80 cm
	1990	Tangents (a) (1)||	27	6 MV	Breast or chest wall	44.0-52.0	2.0	Superior: 2nd costal cartilage<br>Inferior: 1 cm below inframammary fold<br>Medial: midline<br>Lateral: mid-axillary line	Posterior border divergent<br>Opposing symmetrical fields<br>Collimator not angled<br>Isocentric technique
Denmark	1981	Wide Tangents (b) (2)	15	6 MV	Chest wall, IMC	38.9-50.0	1.9-2.0	Superior: 2nd costal cartilage<br>Inferior: 1 cm below inframammary fold<br>Medial: 3 cm from midline contra<br>Lateral: mid-axillary line	Posterior border divergent<br>Half beam blocked superiorly<br>Fixed SSD 100 cm at centre of posterior field edge
	1982	Wide tangents (McWhirter) (b)	11	250 kV	Chest wall	25.4-36.0	1.7-2.5	Superior: 2nd costal cartilage<br>Inferior: 1 cm below inframammary fold<br>Medial: 2 cm from midline contra<br>Lateral: mid-axillary line	Posterior border divergent<br>Opposing symmetrical fields
	1994	Tangents (a) (3)	54	8 MV	Breast	47.5-52.0	1.9-2.2	Superior: 2nd costal cartilage<br>Inferior: 1 cm below inframammary fold<br>Medial: midline<br>Lateral: mid-axillary line	Block defined posterior field border (tapered 1 cm around breast tissue inferiorly)<br>Fixed SSD 100cm at centre of posterior field edge
	1998	Partially wide tangents (i) (4)	12**	8 MV	Breast, IMC	46.0-54.0	1.8-2.0	Superior: 2nd costal cartilage<br>Inferior: 1 cm below inframammary fold<br>Medial: 3 cm from midline contra<br>Lateral: mid-axillary line	Block defined posterior field border (tapered 1 cm around breast tissue below 5th rib)<br>Half-beam-blocked superiorly<br>Fixed SSD 100 cm at centre of posterior field edge

Table continued on next page

Country	Median year	Field arrangement †‡	Number of women	Usual beam energy	Target(s) §	Dose (100%) Gy ¶	Dose/ fraction	Field borders	Comments
Anterior electron or orthovoltage (n=155)									
Sweden	1960	Direct IMC (1-field) (c)	4	170 kV	IMC	14.0-28.0	3.5-4.0	Superior: suprasternal notch<br>Inferior: xiphoid tip<br>Medial: 1 cm from midline contra<br>Lateral: 5 cm from midline	Gantry angle 0°
	1963	Direct IMC (2-fields) (c) (j) (5)	17	12 MeV	IMC	40.0	4.0-5.0	Superior: suprasternal notch<br>Inferior: 14 cm from suprasternal notch<br>Medial: midline<br>Lateral: 4.5 cm from midline	Matching superior and inferior direct anterior beams<br>Typical field size 4.5 cm x 7 cm††<br>SSD 100cm
	1963	Direct chest wall (4 fields) (k)	17	170 kV	Chest wall	20.0-35.0	3.5-4.0	Superior: suprasternal notch<br>Inferior: xiphoid tip<br>Medial: 1 cm from midline contra<br>Lateral: 5 cm from midline	Gantry angle 0°<br>4 x matching direct anterior beams<br>Only the two medial chest wall fields were reconstructed because the other fields were distant from the heart
	1974	Oblique chest wall (d) (6)	21	12 MeV	Chest wall, IMC	44.2-52.5	1.8-2.1	Superior: suprasternal notch<br>Inferior: 1 cm below inframammary fold<br>Medial: midline<br>Lateral: midaxillary line	Gantry angle 35° towards contra side<br>Central bolus (1 cm thick and 8 cm wide)<br>Lower medial corner of the field was blocked<br>Typical field size 14.5 cm x 17 cm††<br>Fixed SSD 120 cm
Denmark	1981	Direct chest wall<br>Lateral thorax, SCF, axilla (n)	15	100 kV	Chest wall	33.0-48.0	2.0-3.0	*Chest wall field*<br>Superior: suprasternal notch<br>Inferior: xiphisternum joint<br>Medial: midline<br>Lateral: 1 cm medial to lateral lung border	Gantry angle 0°<br>Typical field size 12 cm x 10 cm††
				8 MV	Lateral thorax, SCF, axilla	48.0-50.0	2.0-4.2	*Lat thorax, SCF, axilla field*<br>Superior: inferior border C6 vertebra<br>Inferolateral: inframammary fold or 3 cm below scar<br>Inferomedial: 2nd rib<br>Medial superior: 1 cm from midline contra<br>Medial inferior: 1 cm medial to lateral lung<br>Lateral: 3 cm lateral to humeral head	Gantry angle 15° towards ipsilateral side<br>Chest wall block (lateral border 1 cm medial to lateral lung)<br>90% dose at half the AP diameter below humeral head<br>Typical field size 14 cm x 24 cm††<br>Fixed SSD 100 cm
	1982	Direct chest wall<br>Lateral thorax, SCF, axilla (e) (m) (7)	43‡‡	9 MeV	Chest wall	39.8-54.3	2.2-4.0	*Chest wall field*<br>Superior: 2nd rib<br>Inferior: lateral lung border at level of 7th rib<br>Medial: 1 cm from midline contra<br>Lateral: 1 cm medial to lateral lung border	Gantry angle 0°<br>92% of dose delivered at 3cm depth<br>Fixed SSD 100 cm
				8 MV	Lateral thorax, SCF, axilla	40.9-54.7	2.1-4.2	*Lat thorax, SCF, axilla field*<br>Superior: inferior border C6 vertebra<br>Inferolateral: inframammary fold or 3 cm below scar<br>Inferomedial: 2nd rib<br>Medial superior: 1 cm from midline contra<br>Medial inferior: 1 cm medial to lateral lung<br>Lateral: 3 cm lateral to humeral head	Gantry angle 15° towards ipsilateral side<br>Chest wall block (lateral border 1 cm medial to lateral lung)<br>90% dose at half the AP diameter below humeral head<br>Typical field size 14 cm x 24 cm††<br>Fixed SSD 100 cm

Table continued on next page

Country	Median year	Field arrangement †‡	Number of women	Usual beam energy	Target(s) §	Dose (100%) Gy ¶	Dose/ fraction	Field borders	Comments
	1987	Direct IMC<br>Direct chest wall<br>Lateral thorax, SCF, axilla (l)	30§§	9 MeV	IMC	48.3-54.3	2.0-2.4	*IMC field*<br>Superior: 1st costal cartilage<br>Inferior: middle of 5th costal cartilage<br>Medial: 1 cm from midline contra<br>Lateral superior: 5 cm from midline<br>Lateral inferior: 4 cm from midline	Gantry angle 0°<br>85% dose at depth of pleura plus 0.5 cm<br>Fixed SSD 100cm
				6 MeV	Chest wall	48.0-54.3	2.0-2.4	*Chest wall field*<br>Superior: 1st costal cartilage<br>Inferior: inframammary fold or 3 cm below scar<br>Medial: matching IMC field<br>Lateral: 1 cm medial to lateral lung border	Gantry angle 0°<br>85% dose at depth of pleura minus 0.5 cm<br>Fixed SSD 100 cm
				8 MV	Lateral thorax, SCF, axilla	48.0-55.9	2.1-2.4	*Lat thorax, SCF, axilla field*<br>Superior: inferior border C6 vertebra<br>Inferolateral: inframammary fold or 3 cm below scar<br>Inferomedial: 2nd rib<br>Medial superior: 1 cm from midline contra<br>Medial inferior: 1 cm medial to lateral lung<br>Lateral: 3 cm lateral to humeral head	Gantry angle 15° towards ipsilateral side<br>Chest wall block (lateral border 1 cm medial to lateral lung)<br>90% dose at half the AP diameter below humeral head<br>Typical field size 14 cm x 24 cm††<br>Fixed SSD 100 cm
	1991	Oblique chest wall (d)	8	12 MeV	Chest wall	51.6-54.0	2.2-2.4	Superior: suprasternal notch<br>Inferior: 1 cm below inframammary fold<br>Medial: midline<br>Lateral: midaxillary line	Gantry angle 35° towards contra side<br>Central bolus (1 cm thick x 8 cm wide)<br>Lower medial corner blocked<br>Typical field size 14.5 cm x 17 cm††<br>Fixed SSD 120 cm
Anterior megavoltage (n=56)									
Sweden	1969	Oblique IMC<br>Oblique chest wall (f)	17	Co^{60}	IMC	36.0	4.0	*IMC field*<br>Superior: suprasternal notch<br>Inferior: xiphisternum<br>Medial: 4.5 cm from midline contra<br>Lateral: 4.5 cm from midline	Gantry angle 30° towards ipsilateral side<br>SSD 80 cm
				Co^{60}	Chest wall	32.0-36.0	4.0	*Chest wall field*<br>Superior: suprasternal notch<br>Inferior: xiphisternum<br>Medial: matched to IMC field<br>Lateral: 2 cm from skin surface	Gantry angle 30° towards ipsilateral side<br>SSD 80 cm
	1974	Direct bilateral IMC (g)	14	Co^{60}	IMC	36.0-42.0	3.0-4.0	Superior: suprasternal notch<br>Inferior: xiphisternum<br>Medial: 4.5 cm from midline contra<br>Lateral: 4.5 cm from midline ipsilateral	Gantry angle 0°<br>IMC field centred at midline<br>Typical field size 9 cm x 21 cm††<br>SSD 80 cm
	1983	Direct IMC<br>Oblique chest wall (h)	25	Co^{60}	IMC	49.5-52.3	2.0-2.8	*IMC field*<br>Superior: suprasternal notch<br>Inferior: xiphoid tip<br>Medial: midline<br>Lateral: 5.5 cm from midline contra	Gantry angle 0°<br>Typical field size 5.5 cm x 15.5 cm††<br>SSD 80 cm
				9 MeV	Chest wall	47.3-48.5	1.9-2.6	*Chest wall field*<br>Superior: suprasternal notch<br>Inferior: xiphisternum<br>Medial: matched to IMC field<br>Lateral: mid-axillary line	Gantry angle 40° towards contra side<br>Typical field size 9 cm x 21 cm††<br>SSD 100 cm
***Cobalt chain (n=74)* ¶¶**									
Sweden	1963	Cobalt chain long (o)	36	Co^{60}	IMC, SCF	7.0-9.0	3.5	Isocentre 2.5 cm from midline<br>Inferior: 4th intercostal space	3.5 cm x 6 cm overlapping fields along the IMC
	1966	Cobalt chain short (p)	38	Co^{60}	IMC, SCF	7.0-9.0	3.5	Isocentre 2.5 cm from midline<br>Inferior: 7 cm below 4th intercostal space	3.5 cm x 6 cm overlapping fields along the IMC

*Regimens are ordered as in Tables 1,2, and Tables E3,4,7-11. Each technique listed accounts for two regimens, one for left-sided and one for right-sided cancers apart from one direct megavoltage technique (direct bilateral IMC) which was the same for left and right-sided cancers.
Information was collated from radiotherapy charts, radiotherapy protocols and from oncologists and physicists who had delivered them (see references (i)-(vi) below).
†Regimens a-p are illustrated in Fig. 1. and Fig. E1.
‡Regimens 1-7 were reconstructed on 10 CT scans to study the effect of patient anatomy on segment doses (Fig. 3-5).
§Some regimens included separate fields to the SCF and/or axilla but as the doses to the whole heart and to all of the cardiac structures were less than 0.5 Gy, they were not reconstructed. Some regimens included separate field(s) to boost the surgical bed after breast conserving surgery but information was lacking on the location of the surgical bed and heart dose from these fields is likely to be ≤ 1.0 Gy (see Taylor 2007[(vii)]) so these boost fields were not reconstructed.
¶Usual total dose (100%) to the target tissues. For direct regimens this was the Dmax. For tangential regimens this was the dose delivered to the centre of the breast/chest wall apart from orthovoltage tangents where the total dose was the skin dose at the surface of the breast.
||One woman in the category received 36 Gy in 12 fractions.
**This includes 3 women irradiated in Sweden whose radiotherapy was similar to this Danish technique.
††Field size: width (cm) x length (cm).
‡‡This includes 7 women irradiated in Sweden whose radiotherapy was similar to this Danish technique.
§§This includes 3 Swedish women who received techniques similar to this Danish technique.
¶¶For description of cobalt chain see Taylor 2009[(i)].
Abbreviations: kV: kilovoltage, MV: megavoltage; MeV: mega electron-volts, SCF: supraclavicular fossa, IMC: internal mammary chain, contra: contralateral, SSD: source-surface distance, AP: antero-postero, Co^{60}: cobalt 60.

References
(i)Taylor CW, Nisbet A, McGale P, et al. Cardiac doses from Swedish breast cancer radiotherapy since the 1950s. Radiother Oncol 2009;90:127-35.
(ii)Taylor CW, Bronnum D, Darby SC, et al. Cardiac dose estimates from Danish and Swedish breast cancer radiotherapy during 1977-2001. Radiother Oncol. 2011;100:176-83.
(iii)Strender LE, Wallgren A, Arndt J, et al. Adjuvant radiotherapy in operable breast cancer: correlation between dose in internal mammary nodes and prognosis. Int J Radiat Oncol Biol Phys 1981;7:1319-25.
(iv)Overgaard M, Christensen JJ. Postoperative radiotherapy in DBCG during 30 years. Techniques, indications and clinical radiobiological experience. Acta Oncol 2008;47:639-53.
(v)Nielsen HM, Overgaard J, Grau C, Christensen JJ, Overgaard M. Audit of the radiotherapy in the DBCG 82 b&c trials--a validation study of the 1,538 patients randomised to postmastectomy radiotherapy. Radiother Oncol 2005;76:285-92.
(vi)Thomsen MS, Berg M, Nielsen HM, et al. Post-mastectomy radiotherapy in Denmark: from 2D to 3D treatment planning guidelines of The Danish Breast Cancer Cooperative Group. Acta Oncol 2008;47:654-61.
(vii)Taylor CW, Nisbet A, McGale P, Darby SC. Cardiac exposures in breast cancer radiotherapy: 1950s-1990s. Int J Radiat Oncol Biol Phys 2007;69:1484-95.

Table E2. 'Typical-CT' selection

	Mean heart dose (Gy)		Anatomical measurements			
Scan No.	Left tangents, midline divergent (a)(3)*	Left direct chest wall electrons (e)(7)*	Sternal length (cm)	Heart volume (cc)	Chest wall separation (cm)†	Haller index‡
1	1.8	1.9	17.0	781.7	24.3	2.1
2	5.5	3.4	18.0	719.6	22.3	1.8
3	1.2	1.6	16.0	1010.5	21.6	1.9
4	3.7	1.9	19.0	901.7	20.0	2.6
5§	4.0	4.8	17.0	710.5	19.0	1.9
6	3.1	2.9	16.0	836.0	20.9	1.9
7	8.2	5.4	16.0	691.4	19.3	3.4
8	7.6	6.9	19.0	834.4	20.4	2.6
9	0.9	2.0	18.0	826.6	21.0	2.5
10	4.6	3.0	19.0	615.8	18.5	0.5
Correlation of anatomical measurements with mean heart dose estimates¶						
Regimen (a)			*r= -0.06, p=0.87*	*r= -0.37, p=0.27*	*r= -0.30, p=0.40*	*r= 0.52, p=0.13*
Regimen (e)			*r= 0.11, p=0.77*	*r= -0.39, p=0.27*	*r= -0.43, p=0.22*	*r= 0.32, p=0.34*

*Regimens (a) and (e) are illustrated in Fig. 1. For regimen (e) the doses reported here are from the direct electron chest wall beam only, not the the lateral photon chest wall field. These regimens are also numbered (3) and (7) in other tables and figures.

†Chest wall separation: the distance between the midline and the midaxillary line

‡Haller index: ratio of height between anterior spine and posterior sternum to the transverse width of the chest.

§Scan 5 was selected as the 'average CT dataset' because it was the scan with mean heart doses closest to average from both techniques a and e (which were the two most commonly identified left-sided regimens) and was not atypical for any of the anatomical factors examined.

¶The anatomical factors evaluated did not correlate with mean heart dose and no scan had consistently average readings for these measurements.

r= correlation coefficient testing the strength of correlation between mean heart dose and anatomical measurements

p= test for correlation equal to zero

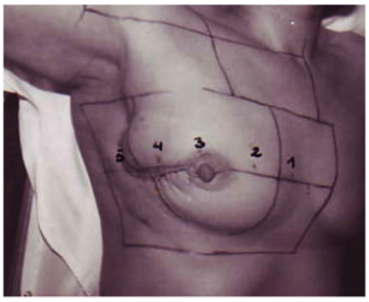

(i) Partially wide tangents

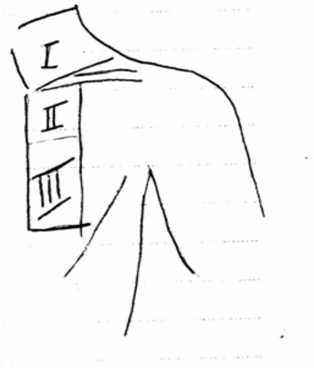

(j) Direct IMC (2-fields)

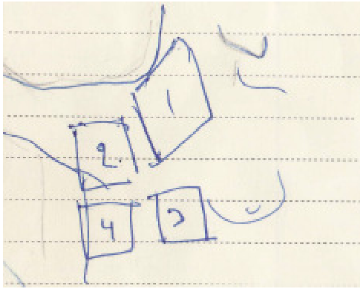

(k) Direct chest wall (4-fields)

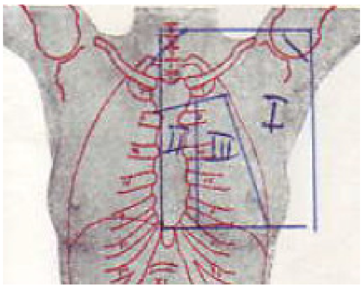

(l) Direct chest wall (2-fields), lateral thorax, SCF, axilla

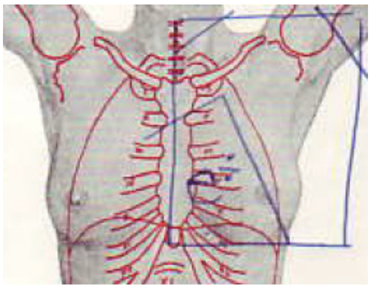

(m) Direct chest wall (1-field), lateral thorax, SCF, axilla

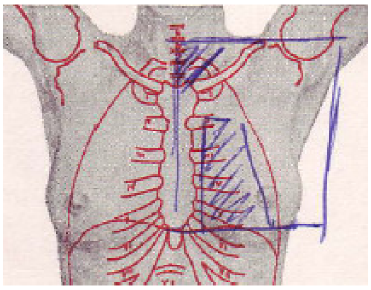

n) Direct chest wall (1-field) lateral thorax, SCF, axilla

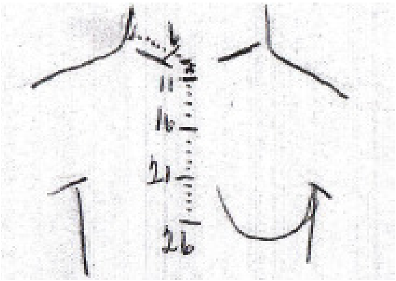

(o) Cobalt chain long

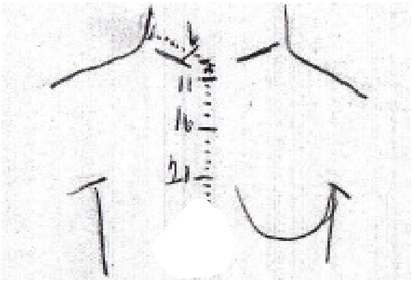

(p) Cobalt chain short

Fig. E1. Radiotherapy regimens used to treat women with breast cancer in Sweden (1958 -2001) or Denmark (1978-2000).

Abbreviations: IMC: internal mammary chain

Table E3. Mean radiation therapy doses to myocardial structures from right-sided breast cancer radiotherapy regimens used in Sweden (1958-2001) or Denmark (1978-2000).

Radiotherapy regimen*						Mean cardiac doses (Gy)†							
							Ventricles:		Left ventricular myocardial segments:				
Country	*Median year*	*Medial border*	*Field arrangement‡§*	*Usual beam energy*	*Dose (100%) Gy¶*	*Whole heart*	*Left*	*Right*	*Apex*	*Lateral*	*Inferior*	*Septal*	*Anterior*
Tangential													
Sweden	1959	Midline	Tangents, divergent (a)	170 kV	10.5	1§	1	1	1	<1	<1	1	1
	1975	6 cm contra	Wide tangents, divergent (b)	Co^{60}	45.0	3.9	1.4	6.5	1.3	0.9	1.1	2.0	1.4
	1982	Midline	Tangents, divergent (a)	Co^{60}	50.0	1.2	0.8	1.4	0.7	0.6	0.8	1.0	0.8
	1990	Midline	Tangents, divergent (a) (1)	6 MV	50.0	0.7	0.2	0.9	0.1	0.1	0.3	0.4	0.2
Denmark	1981	3 cm contra	Wide tangents, divergent (b) (2)	6 MV	40.7	1.2	0.5	1.8	0.4	0.1	0.5	1.0	0.4
	1982	2 cm contra	Wide tangents (McWhirter) (b)	250 kV	36.0	5	3	7	3	1	3	4	3
	1994	Midline	Tangents, block posteriorly (a) (3)	8 MV	48.0	0.4	0.1	0.5	0.1	0.1	0.2	0.2	0.1
	1998	3 cm contra	Partially wide tangents, block posteriorly (i) (4)	8 MV	48.0	0.8	0.2	1.1	0.1	0.1	0.3	0.4	0.2
Anterior electron or orthovoltage													
Sweden	1960	1 cm contra	Direct IMC (1-field) (c)	170 kV	28.0	7	1	6	1	1	5	5	1
	1963	Midline	Direct IMC (2-fields) (c) (j) (5)	12 MeV	40.0	3.1	0.2	2.6	0.1	0.1	0.3	0.4	0.2
	1963	1 cm contra	Direct chest wall (4 fields) (k)	170 kV	20.0	5	1	5	1	1	4	3	1
	1974	1 cm contra	Oblique chest wall (d) (6)	12 MeV	47.8	4.8	0.6	5.4	0.5	0.5	0.6	0.9	0.8
Denmark	1981	Midline	Direct chest wall (1-field)/lat thorax, SCF, axilla (n)	100 kV/8 MV	36.0/50.0	6	1	4	<1	1	2	2	1
	1982	1 cm contra	Direct chest wall (1-field)/lat thorax, SCF, axilla (e) (m) (7)	9 MeV/8 MV	51.8/51.7	3.1	0.4	3.2	0.2	0.3	0.6	0.5	0.4
	1987	1 cm contra	Direct chest wall (2-fields)/lat thorax, SCF, axilla (l)	9 MeV/6 MeV/8 MV	54.0/50.0/54.0	2.4	0.4	1.9	0.2	0.3	0.5	0.5	0.4
	1991	Midline	Oblique chest wall (d)	12 MeV	51.8	5.2	0.7	5.8	0.5	0.5	0.6	1	0.9
Anterior megavoltage													
Sweden	1969	4.5 cm contra	Oblique IMC/oblique chest wall (f)	Co^{60}/Co^{60}	36.0/32.0	11.6	1.8	19.9	0.8	0.9	2.0	3.9	1.2
	1974	4.5 cm contra	Direct bilateral IMC (g) ||	Co^{60}	40.0	21.7	12.8	30.0	1.8	4.4	21.1	24.1	5.8
	1983	Midline	Direct IMC/oblique chest wall (h)	Co^{60}/9 MeV	50.6/48.3	10.4	1.0	7.7	0.4	0.7	1.7	1.3	0.8
Cobalt chain													
Sweden	1963	Midline	Cobalt chain long (overlapping fields) (o)	Co^{60}	7.0	3	1	2	<1	<1	2	2	1
	1966	Midline	Cobalt chain short (overlapping fields) (p)	Co^{60}	7.0	<1	<1	<1	<1	<1	<1	<1	<1

Highlighted tangential regimens are *wide* tangents, others are *midline* tangents. Highlighted anterior electron or orthovoltage regimens are *oblique* electron fields, others include *direct* fields.

*For further details on the radiotherapy regimens see Table E1.

†Mean cardiac doses estimated using manual planning i.e. orthovoltage and cobalt chain are given to nearest Gy.

‡Regimens a-p are illustrated in Fig. 1 and Fig. E1.

§Regimens 1-7 were reconstructed on 5 CT scans to study the effect of patient anatomy on segment doses (Fig. 3-5).

¶Usual total dose (100%) to the target tissues. For direct regimens this was the Dmax. For tangential regimens this was the dose delivered to the centre of the breast/chest wall apart from orthovoltage tangents where the total dose was the skin dose at the surface of the breast.

||Cardiac doses are the same for left-sided and right-sided breast cancer as the same field was used for both.

**For description of cobalt chain see Taylor 2009[i].

Abbreviations: kV: kilovoltage, MV: megavoltage; MeV: mega electron-volts, lat: lateral, SCF: supraclavicular fossa, IMC: internal mammary chain, contra: contralateral, Co^{60}: cobalt 60

(i) Taylor CW, Nisbet A, McGale P, et al. Cardiac doses from Swedish breast cancer radiotherapy since the 1950s. Radiother Oncol 2009;90:127-35.

Table E4. Mean radiation therapy doses to coronary arterial structures from right-sided breast cancer radiotherapy regimens used in Sweden (1958-2001) or Denmark (1978-2000).

Radiotherapy regimen*						Mean coronary artery doses (Gy)†												
	Median	*Medial*			*Dose*	*left*	*left anterior descending*				*right*					*circumflex*		
Country	*year*	*border*	*Field arrangement‡§*	*Usual beam energy*	*(100%) Gy§¶*	*main*	*whole*	*prox*	*mid*	*dist*	*whole*	*prox*	*mid*	*dist*	*pd*	*whole*	*prox*	*dist*
Tangential																		
Sweden	1959	Midline	Tangents, divergent (a)	170 kV	10.5	1	1	1	1	1	2	2	3	3	1	1	1	<1
	1975	6 cm contra	Wide tangents, divergent (b)	Co^{60}	45.0	2.0	2.3	1.8	2.3	2.6	12.1	11.9	32.0	4.0	1.2	1.2	1.4	1.2
	1982	Midline	Tangents, divergent (a)	Co^{60}	50.0	1.1	0.9	0.9	0.9	0.8	1.8	2.1	3.2	1.2	0.7	0.8	0.9	0.8
	1990	Midline	Tangents, divergent (a) (1)	6 MV	50.0	0.5	0.3	0.2	0.3	0.3	1.3	1.6	2.5	0.9	0.4	0.3	0.3	0.3
Denmark	1981	3 cm contra	Wide tangents, divergent (b) (2)	6 MV	40.7	0.9	0.8	0.7	0.7	0.8	3.5	2.6	9.3	1.5	0.8	0.3	0.5	0.3
	1982	2 cm contra	Wide tangents (McWhirter) (b)	250 kV	36.0	4	4	4	4	4	8	10	11	13	4	3	4	3
	1994	Midline	Tangents, block posteriorly (a) (3)	8 MV	48.0	0.3	0.1	0.1	0.1	<0.1	1.0	1.2	2.1	0.6	0.2	0.2	0.2	0.2
	1998	3 cm contra	Partially wide tangents, block posteriorly (i) (4)	8 MV	48.0	0.6	0.2	0.3	0.2	0.2	2.1	2.4	5.0	1.0	0.3	0.3	0.3	0.3
Anterior electron or orthovoltage																		
Sweden	1960	1 cm contra	Direct IMC (1-field) (c)	170 kV	28.0	3	1	2	1	1	12	18	18	21	3	7	3	8
	1963	Midline	Direct IMC (2-fields) (c) (j) (5)	12 MeV	40.0	0.8	0.2	0.3	0.2	<0.1	12.5	21.6	25.7	1.2	0.2	0.4	0.6	0.4
	1963	1 cm contra	Direct chest wall (4 fields) (k)	170 kV	20.0	2	1	1	1	<1	9	13	13	15	2	5	2	5
	1974	1 cm contra	Oblique chest wall (d) (6)	12 MeV	47.8	1.0	1.5	2.3	1.5	0.7	12.4	15.0	26.3	2.6	0.6	0.7	0.7	0.6
Denmark	1981	Midline	Direct chest wall (1-field)/lat thorax, SCF, axilla (n)	100 kV/8 MV	36.0/50.0	4	1	2	1	<1	11	23	24	18	1	4	3	4
	1982	1 cm contra	Direct chest wall (1-field)/lat thorax, SCF, axilla (e)(m)(7)	9 MeV/8 MV	51.8/51.7	0.8	0.3	0.5	0.3	0.2	9.6	12.7	21.4	2.4	1.0	1.0	0.6	1.1
	1987	1 cm contra	Direct chest wall (2-fields)/lat thorax, SCF, axilla (l)	9 MeV/6 MeV/8 MV	54.0/50.0/54.0	0.8	0.4	0.6	0.4	0.2	8.2	12.8	17.1	2.2	0.5	0.8	0.6	0.8
	1991	Midline	Oblique chest wall (d)	12 MeV	51.8	1.0	1.6	2.5	1.6	0.7	13.4	21.1	28.5	2.8	0.6	0.7	0.8	0.7
Anterior megavoltage																		
Sweden	1969	4.5 cm contra	Oblique IMC/oblique chest wall (f)	Co^{60}/Co^{60}	36.0/32.0	4.2	1.2	1.4	1.1	1.1	18.5	28.2	29.5	17.6	3.8	2.2	1.7	2.3
	1974	4.5 cm contra	Direct bilateral IMC (g) ||	Co^{60}	40.0	28.1	4.0	8.2	1.8	2.3	28.0	32.4	33.4	26.0	21.0	21.7	23.9	21.4
	1983	Midline	Direct IMC/oblique chest wall (h)	Co^{60}/9 MeV	50.6/48.3	1.9	0.6	0.8	0.5	0.4	22.8	31.5	42.6	22.5	2.6	2.7	1.3	2.9
Cobalt chain*																		
Sweden	1963	Midline	Cobalt chain long (overlapping fields) (o)	Co^{60}	7.0	1	<1	1	1	<1	7	8	10	3	3	1	1	1
	1966	Midline	Cobalt chain short (overlapping fields) (p)	Co^{60}	7.0	<1	<1	<1	<1	<1	<1	<1	<1	<1	<1	<1	<1	<1

Highlighted tangential regimens are *wide* tangents, others are *midline* tangents. Highlighted anterior electron or orthovoltage regimens are *oblique* electron fields, others include *direct* fields.

*For further details on the radiotherapy regimens see Table E1.

†Mean cardiac doses estimated using manual planning i.e. orthovoltage and cobalt chain are given to nearest Gy.

‡Regimens a-p are illustrated in Fig. 1 and Fig. E1.

§Regimens 1-7 were reconstructed on 5 CT scans to study the effect of patient anatomy on segment doses (Fig. 3-5).

¶Usual total dose (100%) to the target tissues. For direct regimens this was the Dmax. For tangential regimens this was the dose delivered to the centre of the breast/chest wall apart from orthovoltage tangents where the total dose was the skin dose at the surface of the breast.

||Cardiac doses are the same for left-sided and right-sided breast cancer as the same field was used for both.

**For description of cobalt chain see Taylor 2009[i].

Abbreviations: kV: kilovoltage, MV: megavoltage; MeV: mega electron-volts, lat: lateral, SCF: supraclavicular fossa, IMC: internal mammary chain, contra: contralateral, prox: proximal, dist: distal, pd: posterior descending, Co^{60}: cobalt 60

(i) Taylor CW, Nisbet A, McGale P, et al. Cardiac doses from Swedish breast cancer radiotherapy since the 1950s. Radiother Oncol 2009;90:127-35

Table E5. Cardiac segments ordered according to dose received from left breast cancer radiotherapy regimens, comparison of order in typical CT scan with order in nine other scans.

Left breast cancer radiotherapy regimens	Typical CT-scan cardiac segments ordered according to dose received<br>Number with order the same as typical CT-scan/ total number of scans assessed									
**Midline divergent tangents (1)**										
Left ventricle segments	*apex*	*anterior*	*septal*	*lateral*	*inferior*					
	9/9	9/9	9/9	9/9	9/9					
Whole coronary arteries	*ladca*	*rca*	*cflx*							
	9/9	9/9	9/9							
Coronary artery segments	*ladca dist*	*ladca mid*	*ladca prox*	*cflx prox*	*lmca*	*rca pd*	*rca prox*	*rca mid*	*cflx distal*	*rca dist*
	9/9	9/9	8/9	5/9	4/9	4/9	5/9	9/9	9/9	9/9
**Wide divergent tangents (2)**										
Left ventricle segments	*apex*	*anterior*	*septal*	*lateral*	*inferior*					
	9/9	7/9	7/9	9/9	9/9					
Whole coronary arteries	*ladca*	*rca*	*cflx*							
	9/9	9/9	9/9							
Coronary artery segments	*ladca mid*	*ladca dist*	*ladca prox*	*rca pd*	*rca prox*	*cflx prox*	*lmca*	*rca mid*	*cflx dist*	*rca dist*
	1/9	1/9	6/9	6/9	9/9	9/9	8/9	8/9	8/9	8/9
**Midline tangents, block posteriorly (3)**										
Left ventricle segments	*apex*	*anterior*	*septal*	*lateral*	*inferior*					
	9/9	9/9	8/9	8/9	9/9					
Whole coronary arteries	*ladca*	*rca*	*cflx*							
	9/9	7/9	7/9							
Coronary artery segments	*ladca mid*	*ladca dist*	*ladca prox*	*rca pd*	*cflx prox*	*lmca*	*cflx dist*	*rca prox*	*rca mid*	*rca dist*
	2/9	2/9	8/9	6/9	7/9	7/9	6/9	5/9	9/9	9/9
**Partially wide tangents, block posteriorly (4)**										
Left ventricle segments	*apex*	*anterior*	*septal*	*lateral*	*inferior*					
	9/9	7/9	7/9	9/9	9/9					
Whole coronary arteries	*ladca*	*rca*	*cflx*							
	9/9	9/9	9/9							
Coronary artery segments	*ladca mid*	*ladca dist*	*ladca prox*	*rca prox*	*rca mid*	*cflx prox*	*lmca*	*rca pd*	*cflx dist*	*rca dist*
	4/9	4/9	9/9	7/9	6/9	7/9	8/9	5/9	8/9	8/9
**Direct IMC electrons (5)**										
Left ventricle segments	*septal*	*anterior*	*apex*	*lateral*	*inferior*					
	7/9	7/9	7/9	8/9	6/9					
Whole coronary arteries	*rca*	*ladca*	*cflx*							
	3/9	3/9	7/9							
Coronary artery segments	*rca prox*	*ladca prox*	*lmca*	*rca mid*	*ladca mid*	*cflx prox*	*ladca dist*	*cflx dist*	*rca dist*	*rca pd*
	4/9	6/9	7/9	7/9	7/9	8/9	7/9	8/9	9/9	9/9
**Oblique chest wall electrons (6)**										
Left ventricle segments	*apex*	*anterior*	*septal*	*lateral*	*inferior*					
	7/9	7/9	4/9	4/9	9/9					
Whole coronary arteries	*ladca*	rca	*cflx*							
	9/9	7/9	7/9							
Coronary artery segments	*ladca dist*	*ladca mid*	*ladca prox*	rca mid	*cflx prox*	*rca prox*	*lmca*	*cflx dist*	rca pd	rca dist
	5/9	5/9	8/9	2/9	3/9	4/9	8/9	6/9	6/9	8/9
**Direct chest wall electrons (7)**										
Left ventricle segments	*anterior*	*apex*	*septal*	*lateral*	*inferior*					
	7/9	7/9	9/9	9/9	9/9					
Whole coronary arteries	*ladca*	rca	*cflx*							
	9/9	8/9	8/9							
Coronary artery segments	*ladca mid*	*ladca prox*	*ladca dist*	*rca prox*	rca mid	*lmca*	*cflx prox*	*cflx dist*	rca pd	rca dist
	9/9	7/9	5/9	8/9	9/9	9/9	7/9	8/9	8/9	8/9

For further details on radiotherapy regimens 1-7 see Table E1.

Abbreviations: rca: right coronary artery; lmca: left main coronary artery; ladca: left anterior descending coronary artery; cflx: circumflex coronary artery; prox: proximal; dist: distal; pd:posterior descending; IMC: internal mammary chain

Table E6. Cardiac segments ordered according to dose received from right breast cancer radiotherapy regimens, comparison of order in typical CT scan with order in four other scans.

Right breast cancer radiotherapy regimens	Typical CT-scan cardiac segments ordered according to dose received<br>Number with order the same as typical CT-scan/ total number of scans assessed									
**Midline divergent tangents (1)**										
Left ventricle segments	*septal*	*inferior*	*anterior*	*apex*	*lateral*					
	4/4	4/4	4/4	4/4	4/4					
Whole coronary arteries	rca	*ladca*	*cflx*							
	4/4	4/4	4/4							
Coronary artery segments	rca mid	*rca prox*	rca dist	*lmca*	*ladca prox*	*ladca mid*	*cflx prox*	rca pd	*ladca dist*	*cflx dist*
	4/4	4/4	4/4	4/4	4/4	4/4	4/4	4/4	4/4	4/4
**Wide divergent tangents (2)**										
Left ventricle segments	*septal*	*inferior*	*anterior*	*apex*	*lateral*					
	4/4	4/4	4/4	4/4	4/4					
Whole coronary arteries	rca	*ladca*	*cflx*							
	4/4	4/4	4/4							
Coronary artery segments	*rca mid*	*rca prox*	*rca dist*	*lmca*	*ladca prox*	*ladca mid*	*cflx prox*	*rca pd*	*ladca dist*	*cflx dist*
	4/4	4/4	4/4	4/4	4/4	4/4	4/4	4/4	4/4	4/4
**Midline tangents, block posteriorly (3)**										
Left ventricle segments	*septal*	*inferior*	*anterior*	*apex*	*lateral*					
	4/4	4/4	4/4	4/4	4/4					
Whole coronary arteries	rca	*cflx*	*ladca*							
	4/4	4/4	4/4							
Coronary artery segments	*rca mid*	*rca prox*	*rca dist*	*lmca*	*ladca prox*	*ladca mid*	*cflx prox*	*rca pd*	*ladca dist*	*cflx dist*
	4/4	4/4	4/4	4/4	4/4	4/4	4/4	4/4	4/4	4/4
**Partially wide tangents, block posteriorly (4)**										
Left ventricle segments	*septal*	*inferior*	*anterior*	*apex*	*lateral*					
	4/4	4/4	4/4	4/4	4/4					
Whole coronary arteries	rca	*cflx*	*ladca*							
	4/4	3/4	3/4							
Coronary artery segments	*rca mid*	*rca prox*	*rca dist*	*lmca*	*ladca prox*	*ladca mid*	*cflx prox*	*rca pd*	*ladca dist*	*cflx dist*
	4/4	4/4	4/4	4/4	4/4	4/4	4/4	4/4	4/4	4/4
**Direct IMC electrons (5)**										
Left ventricle segments	*septal*	*inferior*	*anterior*	*apex*	*lateral*					
	3/4	4/4	3/4	4/4	4/4					
Whole coronary arteries	rca	*cflx*	*ladca*							
	4/4	4/4	4/4							
Coronary artery segments	*rca mid*	*rca prox*	*rca dist*	*lmca*	*cflx prox*	*ladca prox*	*ladca mid*	*rca pd*	*ladca dist*	*cflx dist*
	4/4	4/4	4/4	4/4	4/4	3/4	4/4	4/4	4/4	3/4
**Oblique chest wall electrons (6)**										
Left ventricle segments	*septal*	*anterior*	*inferior*	*apex*	*lateral*					
	4/4	2/4	2/4	4/4	4/4					
Whole coronary arteries	rca	*ladca*	*cflx*							
	4/4	4/4	4/4							
Coronary artery segments	*rca mid*	*rca prox*	*rca dist*	*ladca mid*	*ladca prox*	*lmca*	*cflx prox*	*rca pd*	*cflx dist*	*ladca dist*
	4/4	4/4	3/4	3/4	3/4	4/4	4/4	4/4	4/4	4/4
**Direct chest wall electrons (7)**										
Left ventricle segments	*septal*	*inferior*	*anterior*	*apex*	*lateral*					
	4/4	4/4	4/4	4/4	4/4					
Whole coronary arteries	rca	*cflx*	*ladca*							
	4/4	4/4	4/4							
Coronary artery segments	rca mid	*rca prox*	rca dist	*lmca*	*ladca prox*	*cflx prox*	*ladca mid*	rca pd	*ladca dist*	*cflx dist*
	3/4	3/4	4/4	4/4	4/4	4/4	4/4	4/4	4/4	4/4

For further details on radiotherapy regimens 1-7 see Table E1.

Abbreviations: rca: right coronary artery; lmca: left main coronary artery; ladca: left anterior descending coronary artery; cflx: circumflex coronary artery; prox: proximal; dist: distal; pd:posterior descending; IMC: internal mammary chain

Table E7. Comparison of doses in this study with previously published estimates for comparable regimens used in Sweden (1958-2001) or Denmark (1978-2000).

Radiotherapy regimen*						Mean heart dose (Gy)†								
						Left-sided regimens				*Right-sided regimens*				
Country	*Median year*	*Medial border*	*Field arrangement‡*	*Usual beam energy*	*Dose (100%) Gy §*	*Duane*	*Taylor*	*Lorenzen*	*Thorsen*	*Duane*	*Taylor*	*Lorenzen*	*Thorsen*	*Likely reason for dose differences*
Tangential														
Sweden	1959	Midline	Tangents (a)	170 kV	10.5	2	2	-	-	1§	1	-	-	
	1975	6cm contra	Wide tangents (b)	Co^{60}	45.0	11.3	16.3	-	-	3.9	6.2	-	-	patient anatomy
	1982	Midline	Tangents (a)	Co^{60}	50.0	5.3	4.7	-	-	1.2	1.2	-	-	
	1990	Midline	Tangents (a)	6 MV	50.0	4.8	2.8	-	-	0.7	2.0	-	-	
Denmark	1981	3cm contra	Wide tangents (b)	6 MV	40.7	9.1	8.5	-	-	1.2	2.7	-	-	
	1982	2cm contra	Wide tangents (McWhirter) (a)	250 kV	36.0	11§	14	-	-	5§	9	-	-	patient anatomy
	1994	Midline	Tangents (a)	8 MV	48.0	4.0	-	2.8	3.4	0.4	-	< 1.0	-	
	1998	3cm contra	Partially wide tangents (i)	8 MV	48.0	4.9	-	3.8	-	0.8	-	< 1.0	0.8	
Anterior electron or orthovoltage														
Sweden	1960	1cm contra	Direct IMC (1-field) (c)	170 kV	28.0	9§	5	-	-	7§	3	-	-	patient anatomy, manual planning uncertainties
	1963	Midline	Direct IMC (2-fields) (c) (j)	12 MeV	40.0	3.7	1.5	-	-	3.1	1.2	-	-	
	1963	1cm contra	Direct chest wall (4 fields) (k)	170 kV	20.0	7§	4	-	-	5§	3	-	-	patient anatomy, manual planning uncertainties
	1974	1cm contra	Oblique chest wall (d)	12 MeV	47.8	9.4	4.1	-	-	4.8	2.0	-	-	patient anatomy, different electron energies available
Denmark	1981	Midline	Direct chest wall (1-field)/lat thorax, SCF, axilla (n)	100 kV/8 MV	36.0/50.0	10§	4	-	-	6§	2	-	-	patient anatomy, manual planning uncertainties
	1982	1cm contra	Direct chest wall (1-field)/lat thorax,SCF, axilla (e) (m)	9 MeV/8 MV	51.8/51.7	6.7	3.0	-	-	3.1	1.9	-	-	patient anatomy, different electron energies available
	1987	1cm contra	Direct chest wall (2-fields)/lat thorax, SCF, axilla (l)	9 MeV/6 MeV/8 MV	54.0/50.0/54.0	5.4	4.5	-	-	2.4	3.0	-	3.5	
	1991	Midline	Oblique chest wall (d)	12 MeV	51.8	9.9	4.4	-	-	5.2	2.0	-	-	patient anatomy, different electron energies available
Anterior megavoltage														
Sweden	1969	4.5cm contra	Oblique IMC/oblique chest wall (f)	Co^{60}/Co^{60}	36.0/32.0	21.9	18.5	-	-	11.6	9.0	-	-	
	1974	4.5cm contra	Direct bilateral IMC (g)	Co^{60}	40.0	21.7	19.5	-	-	21.7	19.5	-	-	
	1983	Midline	Direct IMC/oblique chest wall (h)	Co^{60}/9 MeV	50.6/48.3	20.8	-	-	-	10.4	-	-	-	
Cobalt chain ¶														
Sweden	1963	Midline	Cobalt chain long (overlapping fields) (o)	Co^{60}	7.0	3.9	4.0	-	-	2.6	3.0	-	-	
	1966	Midline	Cobalt chain short (overlapping fields) (p)	Co^{60}	7.0	0.1	0.1	-	-	<0.1	<0.1	-	-	

Where there are differences of > 3 Gy between doses estimated by Duane and Taylor [i,ii] or Lorenzen[iii] or Thorsen[iv], these are highlighted.
"-" Regimen not reconstructed

*For further details on the radiotherapy regimens see Table E1.
†Mean cardiac doses estimated using manual planning i.e. orthovoltage and cobalt chain are given to nearest Gy.
‡Regimens a-p are illustrated in Fig. 1 and Fig. E1.
§Usual total dose (100%) to the target tissues (see Table E1 for dose range). For direct regimens this was the Dmax. For tangential regimens the total dose (100%) was delivered to the centre of the breast or chest wall apart from orthovoltage tangents where the total dose was the skin dose at the surface of the breast.
¶For description of cobalt chain see Taylor 2009[(i)].
Abbreviations: kV: kilovoltage, MV: megavoltage; MeV: mega electron-volts, lat: lateral, SCF: supraclavicular fossa, IMC: internal mammary chain, contra: contralateral, Co^{60}: cobalt 60

(i) Taylor CW, Nisbet A, McGale P, et al. Cardiac doses from Swedish breast cancer radiotherapy since the 1950s. Radiother Oncol 2009;90:127-35.
(ii) Taylor CW, Bronnum D, Darby SC, et al. Cardiac dose estimates from Danish and Swedish breast cancer radiotherapy during 1977-2001. Radiother Oncol. 2011;100:176-83.
(iii) Lorenzen EL, Brink C, Taylor CW, Darby SC, Ewertz M. Uncertainties in estimating heart doses from 2D-tangential breast cancer radiotherapy. Radiother Oncol 2016;119:71-6.
(iv) Thorsen LB, Thomsen MS, Overgaard M, Overgaard J, Offersen BV. Quality assurance of conventional non-CT-based internal mammary lymph node irradiation in a prospective Danish Breast Cancer Cooperative Group trial: the DBCG-IMN study. Acta Oncol 2013;52:1526-34.

Table E8. Mean radiation therapy EQD2 doses to myocardial structures from left-sided breast cancer radiotherapy regimens used in Sweden (1958-2001) or Denmark (1978-2000)

Radiotherapy regimen*							Mean cardiac EQD2† doses (Gy_2)‡							
								Ventricles:		Left ventricular myocardial segments:				
Country	*Median year*	*Medial border*	*Field arrangement§¶*	*Usual beam energy*	*Dose (100%) Gy||*	*No. of fractions*	*Whole heart*	*Left*	*Right*	*Apex*	*Lateral*	*Inferior*	*Septal*	*Anterior*
Tangential														
Sweden	1959	Midline	Tangents, divergent (a)	170 kV**	10.5	3	2	.	.	.	.	.	.	.
	1975	6 cm contra	Wide tangents, divergent (b)	Co^{60}	45.0	26	8.6	10.4	14.1	30.3	4.2	1.0	12.3	14.6
	1982	Midline	Tangents, divergent (a)	Co^{60}	50.0	25	3.8	5.7	3.0	28.6	1.7	0.6	3.4	6.5
	1990	Midline	Tangents, divergent (a) (1)	6 MV	50.0	25	3.8	5.8	2.4	34.2	1.2	0.5	2.8	5.7
Denmark	1981	3 cm contra	Wide tangents, divergent (b) (2)	6 MV	40.7	22	7.8	10.8	11.1	37.0	4.2	0.7	10.9	16.2
	1982	2 cm contra	Wide tangents (McWhirter) (b)	250 kV**	36.0	20	8	.	.	.	.	.	.	.
	1994	Midline	Tangents, block posteriorly (a) (3)	8 MV	48.0	24	3.1	4.7	1.9	28.2	1.1	0.3	2.0	5.6
	1998	3 cm contra	Partially wide tangents, block posteriorly (i) (4)	8 MV	48.0	24	3.7	4.2	4.3	18.8	1.4	0.4	2.9	8.6
Anterior electron or orthovoltage														
Sweden	1960	1 cm contra	Direct IMC (1-field) (c)	170 kV**	28.0	7	11	.	.	.	.	.	.	.
	1963	Midline	Direct IMC (2-fields) (c) (j) (5)	12 MeV	40.0	10	3.6	0.5	8.4	0.3	0.2	0.2	1.3	0.8
	1963	1 cm contra	Direct chest wall (4 fields) (k)	170 kV**	20.0	5	8	.	.	.	.	.	.	.
	1974	1 cm contra	Oblique chest wall (d) (6)	12 MeV	47.8	26	6.9	6.2	10.7	22.9	3.2	0.4	6.1	14.1
Denmark	1981	Midline	Direct chest wall (1-field)/lat thorax, SCF, axilla (n)	100 kV**/8 MV††	36.0/50.0	12/ 12	.	.	.	.	.	.	.	.
	1982	1 cm contra	Direct chest wall (1-field)/lat thorax, SCF, axilla (e) (m) (7)	9 MeV/8 MV	51.8/51.7	24/ 24	4.9	2.9	7.0	6.6	1.7	1.1	2.5	10.5
	1987	1 cm contra	Direct chest wall (2-fields)/lat thorax, SCF, axilla (l)	9 MeV/6 MeV/8 MV	54.0/50.0/54.0	25/25/25	3.6	1.7	5.6	2.2	1.8	1.3	1.7	2.8
	1991	Midline	Oblique chest wall (d)	12 MeV	51.8	24	8.1	7.3	12.6	27.5	3.6	0.4	7.1	16.7
Anterior megavoltage														
Sweden	1969	4.5 cm contra	Oblique IMC/oblique chest wall (f)	Co^{60}/Co^{60}	36.0/32.0	9/9	23.1	30.3	42.7	41.1	23.1	21.5	36.5	31.9
	1974	4.5 cm contra	Direct bilateral IMC (g) ‡‡	Co^{60}	40.0	10	25.5	13.1	37.9	1.1	3.3	22.0	27.7	4.8
	1983	Midline	Direct IMC/oblique chest wall (h)	Co^{60}/9 MeV	50.6/48.3	23/23	19.7	11.9	22.8	3.6	3.8	12.8	22.7	9.2
Cobalt chain §§														
Sweden	1963	Midline	Cobalt chain long (overlapping fields) (o)	Co^{60}**	7.0	1	6	.	.	.	.	.	.	.
	1966	Midline	Cobalt chain short (overlapping fields) (p)	Co^{60}**	7.0	1	<1	.	.	.	.	.	.	.

Highlighted tangential regimens are *wide* tangents, others are *midline* tangents. Highlighted anterior electron or orthovoltage regimens are *oblique* electron fields, others include *direct* fields.

*For further details on the radiotherapy regimens see Table E1.

†EQD2 = nd(d + α/β)/(2 + α/β); EQD2: equivalent dose in 2Gy per fraction; n: number of fractions; d: dose per fraction; α/β: fractionation sensitivity 2 Gy.

‡Mean cardiac doses estimated using manual planning i.e. orthovoltage and cobalt chain are given to nearest Gy.

§Regimens a-p are illustrated in Fig. 1 and Fig. E1.

¶Regimens 1-7 were reconstructed on 10 CT scans to study the effect of patient anatomy on segment doses (Fig. 3-5).

||Usual total dose (100%) to the target tissues. For direct regimens this was the Dmax. For tangential regimens this was the dose delivered to the centre of the breast or chest wall apart from orthovoltage tangents where the total dose was the skin dose at the surface of the breast.

**EQD2 doses were not estimated for the cardiac substructures because of uncertainties in manual planning.

††EQD2 doses were not calculated as it was not possible to combine the DVH for the orthovoltage field generated using manual planning with the DVH for the megavoltage field generated using CT-planning.

‡‡Cardiac doses are the same for left-sided and right-sided breast cancer as the same field was used for both.

§§For description of cobalt chain see Taylor 2009[i].

Abbreviations: kV: kilovoltage, MV: megavoltage; MeV: mega electron-volts, lat: lateral, SCF: supraclavicular fossa, IMC: internal mammary chain, contra: contralateral, Co^{60}: cobalt 60.

(i) Taylor CW, Nisbet A, McGale P, et al. Cardiac doses from Swedish breast cancer radiotherapy since the 1950s. Radiother Oncol 2009;90:127-35.

Table E9. Mean radiation therapy EQD2 doses to coronary arterial structures from left-sided breast cancer radiotherapy regimens used in Sweden (1958-2001) or Denmark (1978-2000).

Radiotherapy regimen*							Mean coronary artery EQD2† doses (Gy_2)												
	Median	*Medial*			*Dose*	*No. of*	*left*	*left anterior descending*				*right*					*circumflex*		
Country	*year*	*border*	*Field arrangement‡§*	*Usual beam energy*	*(100%) Gy¶*	*fractions*	*main*	*whole*	*prox*	*mid*	*dist*	*whole*	*prox*	*mid*	*dist*	*pd*	*whole*	*prox*	*dist*
Tangential																			
Sweden	1959	Midline	Tangents, divergent (a)	170 kV||	10.5	3	.	.	.	.	.	.	.	.	.	.	.	.	.
	1975	6 cm contra	Wide tangents, divergent (b)	Co^{60}	45.0	26	2.0	30.7	22.8	34.9	33.8	3.4	4.6	4.8	1.0	3.2	0.9	1.6	0.8
	1982	Midline	Tangents, divergent (a)	Co^{60}	50.0	25	1.0	28.2	7.6	38.5	37.6	0.8	0.9	0.8	0.6	0.9	0.7	0.9	0.6
	1990	Midline	Tangents, divergent (a) (1)	6 MV	50.0	25	0.8	32.0	6.0	44.5	44.3	0.6	0.7	0.6	0.4	0.7	0.5	0.8	0.4
Denmark	1981	3 cm contra	Wide tangents, divergent (b) (2)	6 MV	40.7	22	0.9	34.5	22.9	40.7	38.7	1.4	1.2	1.1	0.6	2.4	0.6	0.9	0.5
	1982	2 cm contra	Wide tangents (McWhirter) (b)	250 kV||	36.0	20	.	.	.	.	.	.	.	.	.	.	.	.	.
	1994	Midline	Tangents, block posteriorly (a) (3)	8 MV	48.0	24	0.6	31.2	6.0	45.0	41.4	0.4	0.5	0.4	0.2	0.5	0.3	0.6	0.3
	1998	3 cm contra	Partially wide tangents, block posteriorly (i) (4)	8 MV	48.0	24	1.1	31.4	15.7	48.0	29.2	1.0	1.4	1.4	0.3	0.6	0.4	0.9	0.3
Anterior electron or orthovoltage																			
Sweden	1960	1 cm contra	Direct IMC (1-field) (c)	170 kV||	28.0	7	.	.	.	.	.	.	.	.	.	.	.	.	.
	1963	Midline	Direct IMC (2-fields) (c) (j) (5)	12 MeV	40.0	10	2.8	2.2	4.8	1.7	0.3	3.8	11.2	2.5	0.2	0.1	0.4	1.0	0.3
	1963	1 cm contra	Direct chest wall (4 fields) (k)	170 kV||	20.0	5	.	.	.	.	.	.	.	.	.	.	.	.	.
	1974	1 cm contra	Oblique chest wall (d) (6)	12 MeV	47.8	26	4.9	28.7	24.1	30.8	30.8	3.7	5.8	7.1	0.5	0.8	0.8	2.7	0.5
Denmark	1981	Midline	Direct chest wall (1-field)/lat thorax, SCF, axilla (n)	100 kV||/8 MV**	36.0/50.0	12/ 12	.	.	.	.	.	.	.	.	.	.	.	.	.
	1982	1 cm contra	Direct chest wall (1-field)/lat thorax, SCF, axilla (e) (m) (7)	9 MeV/8 MV	51.8/51.7	24/ 24	2.3	28.1	32.4	45.6	6.2	4.2	7.6	6.9	1.4	0.4	4.2	2.0	1.4
	1987	1 cm contra	Direct chest wall (2-fields)/lat thorax, SCF, axilla (l)	9 MeV/6 MeV/8 MV	54.0/50.0/54.0	25/25/25	1.9	9.1	9.8	12.2	4.8	3.5	7.1	4.4	1.0	0.7	1.5	1.8	1.4
	1991	Midline	Oblique chest wall (d)	12 MeV	51.8	24	5.6	34.7	28.9	37.3	37.3	6.5	10.2	12.5	1.1	1.5	1.6	5.3	1.0
Anterior megavoltage																			
Sweden	1969	4.5 cm contra	Oblique IMC/oblique chest wall (f)	Co^{60}/Co^{60}	36.0/32.0	9/9	35.0	43.9	40.7	44.9	45.5	35.7	46.7	46.9	21.3	25.3	20.9	28.2	19.7
	1974	4.5 cm contra	Direct bilateral IMC (g)††	Co^{60}	40.0	10	33.7	3.0	6.9	1.0	1.3	34.6	42.5	44.6	30.1	22.7	22.6	26.2	22.2
	1983	Midline	Direct IMC/oblique chest wall (h)	Co^{60}/9 MeV	50.6/48.3	23/23	28.4	23.2	29.2	19.2	18.8	5.6	11.8	0.2	9.0	3.0	16.9	24.3	15.7
Cobalt chain ‡‡																			
Sweden	1963	Midline	Cobalt chain long (overlapping fields) (o)	Co^{60}||	7.0	1	.	.	.	.	.	.	.	.	.	.	.	.	.
	1966	Midline	Cobalt chain short (overlapping fields) (p)	Co^{60}||	7.0	1	.	.	.	.	.	.	.	.	.	.	.	.	.

Highlighted tangential regimens are *wide* tangents, others are *midline* tangents. Highlighted anterior electron or orthovoltage regimens are *oblique* electron fields, others include *direct* fields.

*For further details on the radiotherapy regimens see Table E1.

†EQD2 = nd(d + α/β)/(2 + α/β); EQD2: equivalent dose in 2Gy per fraction; n: number of fractions; d: dose per fraction; α/β: fractionation sensitivity 2 Gy.

‡Regimens a-p are illustrated in Fig. 1 and Fig. E1.

§Regimens 1-7 were reconstructed on 10 CT scans to study the effect of patient anatomy on segment doses (Fig. 3-5)

¶Usual total dose (100%) to the target tissues. For direct regimens this was the Dmax. For tangential regimens this was the dose delivered to the centre of the breast or chest wall apart from orthovoltage tangents where the total dose was the skin dose at the surface of the breast.

||EQD2 doses were not estimated for the cardiac substructures because of uncertainties in manual planning.

**EQD2 doses were not calculated as it was not possible to combine the DVH for the orthovoltage field generated using manual planning with the DVH for the megavoltage field generated using CT-planning.

††Cardiac doses are the same for left-sided and right-sided breast cancer as the same field was used for both.

‡‡For description of cobalt chain see Taylor 2009[i].

Abbreviations: kV: kilovoltage, MV: megavoltage; MeV: mega electron-volts, lat: lateral, SCF: supraclavicular fossa, IMC: internal mammary chain, contra: contralateral, prox: proximal, dist: distal, pd: posterior descending, Co^{60}: cobalt 60.

(i) Taylor CW, Nisbet A, McGale P, et al. Cardiac doses from Swedish breast cancer radiotherapy since the 1950s. Radiother Oncol 2009;90:127-35.

Table E10. Mean radiation therapy EQD2 doses to myocardial structures from right-sided breast cancer radiotherapy regimens used in Sweden (1958-2001) or Denmark (1978-2000).

Radiotherapy regimen*							Mean cardiac EQD2† doses (Gy$_2$)‡							
								Ventricles:		*Left ventricular myocardial segments:*				
Country	*Median year*	*Medial border*	*Field arrangement§¶*	*Usual beam energy*	*Dose (100%) Gy||*	*No. of fractions*	*Whole heart*	*Left*	*Right*	*Apex*	*Lateral*	*Inferior*	*Septal*	*Anterior*
Tangential														
Sweden	1959	Midline	Tangents, divergent (a)	170 kV**	10.5	3	1	.	.	.	.	.	.	.
	1975	6 cm contra	Wide tangents, divergent (b)	Co^{60}	45.0	26	2.4	0.7	4.0	0.7	0.5	0.6	1.0	0.7
	1982	Midline	Tangents, divergent (a)	Co^{60}	50.0	25	0.6	0.4	0.7	0.3	0.3	0.4	0.5	0.4
	1990	Midline	Tangents, divergent (a) (1)	6 MV	50.0	25	0.4	0.1	0.5	0.1	0.0	0.2	0.2	0.1
Denmark	1981	3 cm contra	Wide tangents, divergent (b) (2)	6 MV	40.7	22	0.7	0.3	0.9	0.2	0.1	0.2	0.5	0.2
	1982	2 cm contra	Wide tangents (McWhirter) (b)	250 kV**	36.0	20	3	.	.	.	.	.	.	.
	1994	Midline	Tangents, block posteriorly (a) (3)	8 MV	48.0	24	0.2	0.1	0.3	<0.1	<0.1	0.1	0.1	<0.1
	1998	3 cm contra	Partially wide tangents, block posteriorly (i) (4)	8 MV	48.0	24	0.4	0.1	0.6	<0.1	<0.1	0.1	0.2	0.1
Anterior electron or orthovoltage														
Sweden	1960	1 cm contra	Direct IMC (1-field) (c)	170 kV**	28.0	7	14	.	.	.	.	.	.	.
	1963	Midline	Direct IMC (2-fields) (c) (j) (5)	12 MeV	40.0	10	2.9	0.1	1.9	0.0	0.1	0.2	0.2	0.1
	1963	1 cm contra	Direct chest wall (4 fields) (k)	170 kV**	20.0	5	10	.	.	.	.	.	.	.
	1974	1 cm contra	Oblique chest wall (d) (6)	12 MeV	47.8	26	3.3	0.3	3.3	0.2	0.2	0.3	0.5	0.4
Denmark	1981	Midline	Direct chest wall (1-field)/lat thorax, SCF, axilla (n)	100 kV**/8 MV††	36.0/50.0	12/ 12	.	.	.	.	.	.	.	.
	1982	1 cm contra	Direct chest wall (1-field)/lat thorax, SCF, axilla (e) (m) (7)	9 MeV/8 MV	51.8/51.7	24/ 24	2.1	0.2	2.0	0.1	0.1	0.3	0.3	0.2
	1987	1 cm contra	Direct chest wall (2-fields)/lat thorax, SCF, axilla (l)	9 MeV/6 MeV/8 MV	54.0/50.0/54.0	25/25/25	1.5	0.2	1.1	0.1	0.2	0.3	0.2	0.2
	1991	Midline	Oblique chest wall (d)	12 MeV	51.8	24	3.7	0.3	3.7	0.3	0.3	0.3	0.5	0.4
Anterior megavoltage														
Sweden	1969	4.5 cm contra	Oblique IMC/oblique chest wall (f)	Co^{60}/Co^{60}	36.0/32.0	9/9	12.1	0.3	27.1	<0.1	0.1	0.3	1.3	0.1
	1974	4.5 cm contra	Direct bilateral IMC (g) ‡‡	Co^{60}	40.0	10	25.5	13.1	37.9	1.1	3.3	22.0	27.7	4.8
	1983	Midline	Direct IMC/oblique chest wall (h)	Co^{60}/9 MeV	50.6/48.3	23/23	9.8	<0.1	3.7	<0.1	<0.1	0.1	<0.1	<0.1
Cobalt chain §§														
Sweden	1963	Midline	Cobalt chain long (overlapping fields) (o)	Co^{60}**	7.0	1	3	.	.	.	.	.	.	.
	1966	Midline	Cobalt chain short (overlapping fields) (p)	Co^{60}**	7.0	1	<1	.	.	.	.	.	.	.

Highlighted tangential regimens are *wide* tangents, others are *midline* tangents. Highlighted anterior electron or orthovoltage regimens are *oblique* electron fields, others include *direct* fields.

*For further details on the radiotherapy regimens see Table E1

†EQD2 = nd(d + α/β)/(2 + α/β); EQD2: equivalent dose in 2Gy per fraction; n: number of fractions; d: dose per fraction; α/β: fractionation sensitivity 2 Gy.

‡Mean cardiac doses estimated using manual planning i.e. orthovoltage and cobalt chain are given to nearest Gy.

§Regimens a-p are illustrated in Fig. 1 and Fig. E1.

¶Regimens 1-7 were reconstructed on 10 CT scans to study the effect of patient anatomy on segment doses (Fig. 3-5)

||Usual total dose (100%) to the target tissues. For direct regimens this was the Dmax. For tangential regimens this was the dose delivered to the centre of the breast or chest wal apart from orthovoltage tangents where the total dose was the skin dose at the surface of the breast.

**EQD2 doses were not estimated for the cardiac substructures because of uncertainties in manual planning

††EQD2 doses were not calculated as it was not possible to combine the DVH for the orthovoltage field generated using manual planning with the DVH for the megavoltage field generated using CT-planning.

‡‡Cardiac doses are the same for left-sided and right-sided breast cancer as the same field was used for both.

§§For description of cobalt chain see Taylor 2009[(i)].

Abbreviations: kV: kilovoltage, MV: megavoltage; MeV: mega electron-volts, lat: lateral, SCF: supraclavicular fossa, IMC: internal mammary chain, contra: contralateral, Co^{60}: cobalt 60.

(i) Taylor CW, Nisbet A, McGale P, et al. Cardiac doses from Swedish breast cancer radiotherapy since the 1950s. Radiother Oncol 2009;90:127-35.

Table E11. Mean radiation therapy EQD2 doses to coronary arterial structures from right-sided breast cancer radiotherapy regimens used in Sweden (1958-2001) or Denmark (1978-2000).

Radiotherapy regimen*							Mean coronary artery EQD2† doses (Gy_2)												
	Median	*Medial*			*Dose*	*No. of*	*left*	*left anterior descending*				*right*					*circumflex*		
Country	*year*	*border*	*Field arrangement‡§*	*Usual beam energy*	*(100%) Gy¶*	*fractions*	*main*	*whole*	*prox*	*mid*	*dist*	*whole*	*prox*	*mid*	*dist*	*pd*	*whole*	*prox*	*dist*
Tangential																			
Sweden	1959	Midline	Tangents, divergent (a)	170 kV||	10.5	3	.	.	.	.	.	.	.	.	.	.	.	.	.
	1975	6 cm contra	Wide tangents, divergent (b)	Co^{60}	45.0	26	1.0	1.2	0.9	1.2	1.4	9.1	7.8	25.9	2.5	0.6	0.6	0.7	0.6
	1982	Midline	Tangents, divergent (a)	Co^{60}	50.0	25	0.6	0.5	0.5	0.5	0.4	0.9	1.1	1.7	0.6	0.4	0.4	0.4	0.4
	1990	Midline	Tangents, divergent (a) (1)	6 MV	50.0	25	0.3	0.1	0.1	0.1	0.1	0.7	0.8	1.3	0.5	0.2	0.2	0.1	0.2
Denmark	1981	3 cm contra	Wide tangents, divergent (b) (2)	6 MV	40.7	22	0.5	0.4	0.3	0.4	0.4	2.1	1.4	5.9	0.8	0.4	0.2	0.3	0.2
	1982	2 cm contra	Wide tangents (McWhirter) (b)	250 kV||	36.0	20	.	.	.	.	.	.	.	.	.	.	.	.	.
	1994	Midline	Tangents, block posteriorly (a) (3)	8 MV	48.0	24	0.2	<0.1	<0.1	<0.1	<0.1	0.5	0.6	1.1	0.3	0.1	0.1	0.1	0.1
	1998	3 cm contra	Partially wide tangents, block posteriorly (i) (4)	8 MV	48.0	24	0.3	0.1	0.1	0.1	0.1	1.1	1.3	2.8	0.5	0.1	0.1	0.2	0.1
Anterior electron or orthovoltage																			
Sweden	1960	1 cm contra	Direct IMC (1-field) (c)	170 kV||	28.0	7	.	.	.	.	.	.	.	.	.	.	.	.	.
	1963	Midline	Direct IMC (2-fields) (c) (j) (5)	12 MeV	40.0	10	0.4	0.1	0.2	0.1	<0.1	14.3	23.3	30.8	0.7	0.1	1.2	0.3	0.2
	1963	1 cm contra	Direct chest wall (4 fields) (k)	170 kV||	20.0	5	.	.	.	.	.	.	.	.	.	.	.	.	.
	1974	1 cm contra	Oblique chest wall (d) (6)	12 MeV	47.8	26	0.5	0.8	1.2	0.8	0.4	9.0	13.7	19.9	1.5	0.3	0.3	0.4	0.3
Denmark	1981	Midline	Direct chest wall (1-field)/lat thorax, SCF, axilla (n)	100 kV||/8 MV**	36.0/50.0	12/ 12	.	.	.	.	.	.	.	.	.	.	.	.	.
	1982	1 cm contra	Direct chest wall (1-field)/lat thorax, SCF, axilla (e) (m) (7)	9 MeV/8 MV	51.8/51.7	24/ 24	0.4	0.2	0.3	0.2	0.1	7.0	9.0	16.1	1.3	0.5	0.5	0.3	0.6
	1987	1 cm contra	Direct chest wall (2-fields)/lat thorax, SCF, axilla (l)	9 MeV/6 MeV/8 MV	54.0/50.0/54.0	25/25/25	0.4	0.2	0.3	0.2	0.1	5.8	8.7	12.0	1.1	0.3	0.4	0.3	0.4
	1991	Midline	Oblique chest wall (d)	12 MeV	51.8	24	0.5	0.8	1.3	0.8	0.4	10.3	15.6	22.8	1.7	0.3	0.4	0.4	0.4
Anterior megavoltage																			
Sweden	1969	4.5 cm contra	Oblique IMC/oblique chest wall (f)	Co^{60}/Co^{60}	36.0/32.0	9/9	1.3	0.1	0.1	0.1	0.1	26.8	44.4	48.4	19.7	1.2	0.3	0.2	0.4
	1974	4.5 cm contra	Direct bilateral IMC (g)††	Co^{60}	40.0	10	33.7	3.0	6.9	1.0	1.3	34.6	42.5	44.6	30.1	22.7	22.6	26.2	22.2
	1983	Midline	Direct IMC/oblique chest wall (h)	Co^{60}/9 MeV	50.6/48.3	23/23	0.1	<0.1	<0.1	<0.1	<0.1	17.4	22.0	39.5	13.0	0.3	0.2	<0.1	0.3
Cobalt chain ‡‡																			
Sweden	1963	Midline	Cobalt chain long (overlapping fields) (o)	Co^{60}||	7.0	1	.	.	.	.	.	.	.	.	.	.	.	.	.
	1966	Midline	Cobalt chain short (overlapping fields) (p)	Co^{60}||	7.0	1	.	.	.	.	.	.	.	.	.	.	.	.	.

Highlighted tangential regimens are *wide* tangents, others are *midline* tangents. Highlighted anterior electron or orthovoltage regimens are *oblique* electron fields, others include *direct* fields.

*For further details on the radiotherapy regimens see Table E1.

†EQD2 = nd(d + α/β)/(2 + α/β); EQD2: equivalent dose in 2Gy per fraction; n: number of fractions; d: dose per fraction; α/β: fractionation sensitivity 2 Gy.

‡Regimens a-p are illustrated in Fig. 1 and Fig. E1.

§Regimens 1-7 were reconstructed on 10 CT scans to study the effect of patient anatomy on segment doses (Fig. 3-5)

¶Usual total dose (100%) to the target tissues. For direct regimens this was the Dmax. For tangential regimens this was the dose delivered to the centre of the breast or chest wall apart from orthovoltage tangents where the total dose was the skin dose at the surface of the breast.

||EQD2 doses were not estimated for the cardiac substructures because of uncertainties in manual planning.

**EQD2 doses were not calculated as it was not possible to combine the DVH for the orthovoltage field generated using manual planning with the DVH for the megavoltage field generated using CT-planning.

††Cardiac doses are the same for left-sided and right-sided breast cancer as the same field was used for both.

‡‡For description of cobalt chain see Taylor 2009[i].

Abbreviations: kV: kilovoltage, MV: megavoltage; MeV: mega electron-volts, lat: lateral, SCF: supraclavicular fossa, IMC: internal mammary chain, contra: contralateral, prox: proximal, dist: distal, pd: posterior descending, Co^{60}: cobalt 60.

(i) Taylor CW, Nisbet A, McGale P, et al. Cardiac doses from Swedish breast cancer radiotherapy since the 1950s. Radiother Oncol 2009;90:127-35.
